# Chemo-drugs in cell microparticles reset antitumor activity of macrophages by activating lysosomal P450 and nuclear hnRNPA2B1

**DOI:** 10.1038/s41392-022-01212-7

**Published:** 2023-01-20

**Authors:** Keke Wei, Huafeng Zhang, Shuaishuai Yang, Yuxiao Cui, Bingxia Zhang, Jincheng Liu, Liang Tang, Yaoyao Tan, Simin Liu, Shiqi Chen, Wu Yuan, Xiao Luo, Chen Chen, Fei Li, Junwei Liu, Jie Chen, Pingwei Xu, Jiadi Lv, Ke Tang, Yi Zhang, Jingwei Ma, Bo Huang

**Affiliations:** 1 Department of Immunology, Tongji Medical College, Huazhong University of Science & Technology, Wuhan, 430030 China; 2 Department of Pathology, School of Basic Medicine, Tongji Medical College, Huazhong University of Science & Technology, Wuhan, 430030 China; 3 Department of Biochemistry & Molecular Biology, Tongji Medical College, Huazhong University of Science & Technology, Wuhan, 430030 China; 4 Cardiovascular Surgery, Union Hospital, Huazhong University of Science & Technology, Wuhan, 430071 China; 5 Department of Immunology & National Key Laboratory of Medical Molecular Biology, Institute of Basic Medical Sciences, Chinese Academy of Medical Sciences (CAMS) & Peking Union Medical College, Beijing, 100005 China; 6 Translational Medicine Laboratory, First Affiliated Hospital of Wenzhou Medical University, Wenzhou, Zhejiang 325035 China; 7 Biotherapy Center and Cancer Center, The First Affiliated Hospital of Zhengzhou University, Zhengzhou, 450052 China

**Keywords:** Cancer therapy, Cancer therapy, Immunotherapy

## Abstract

Macrophages in tumors (tumor-associated macrophages, TAMs), a major population within most tumors, play key homeostatic functions by stimulating angiogenesis, enhancing tumor cell growth, and suppressing antitumor immunity. Resetting TAMs by simple, efficacious and safe approach(s) is highly desirable to enhance antitumor immunity and attenuate tumor cell malignancy. Previously, we used tumor cell-derived microparticles to package chemotherapeutic drugs (drug-MPs), which resulted in a significant treatment outcome in human malignant pleural effusions via neutrophil recruitments, implicating that drug-MPs might reset TAMs, considering the inhibitory effects of M2 macrophages on neutrophil recruitment and activation. Here, we show that drug-MPs can function as an antitumor immunomodulator by resetting TAMs with M1 phenotype and IFN-β release. Mechanistically, drug molecules in tumor MPs activate macrophage lysosomal P450 monooxygenases, resulting in superoxide anion formation, which further amplifies lysosomal ROS production and pH value by activating lysosomal NOX2. Consequently, lysosomal Ca^2+^ signaling is activated, thus polarizing macrophages towards M1. Meanwhile, the drug molecules are delivered from lysosomes into the nucleus where they activate DNA sensor hnRNPA2B1 for IFN-β production. This lysosomal-nuclear machinery fully arouses the antitumor activity of macrophages by targeting both lysosomal pH and the nuclear innate immunity. These findings highlight that drug-MPs can act as a new immunotherapeutic approach by revitalizing antitumor activity of macrophages. This mechanistic elucidation can be translated to treat malignant ascites by drug-MPs combined with PD-1 blockade.

## Introduction

Tumor-associated macrophages (TAMs) are the most abundant tumor-infiltrating immune cells in the tumor microenvironment.^1^ A high density of TAMs infiltration is strongly associated with poor prognosis in tumor patients.^2^ Increasing preclinical and clinical studies suggest that TAMs are considered pivotal for tumor therapy. TAMs are generally categorized into two functional subtypes, namely classical activated macrophages (M1) and alternatively activated macrophages (M2). Both M1 and M2 macrophages are highly plastic and thus can be converted into each other upon tumor microenvironment changes or therapeutic interventions.^3^ Although TAM depletion-based approaches have been developed,^4–6^ how to switch the paradigm from depleting protumor M2 TAMs to reverting M2 to M1 antitumor ones represents a strategy in the next generation of tumor immunotherapy.

Phagocytosing exogenous materials and disposing of the cargo in lysosomes are essential for the function of macrophages, which relies on a low lysosomal pH. Vacuolar-type H^+^-ATPase pumps protons into the lumen, thus decreasing lysosomal pH;^7^ in contrast, lysosomal enzymes continually degrade materials at the expense of protons, thus increasing the pH. Besides, lysosomal NADPH oxidase 2 (NOX2) and the P450 system can cause the protons consumption via ROS generation by catalyzing the transfer of the electron to molecular oxygen.^8–10^ Of note, the lysosomal pH regulation is related to macrophage polarization. Increases in lysosomal pH can reset TAMs towards an M1 antitumor phenotype,^11,12^ in contrast, decreases in the pH biases the M2 polarization.^13^ In addition, lysosomal pH is also related to the migration of lysosomes.^14–16^ Upon increases in lysosomal pH, lysosomes recruit small GTPase Rab7, which further recruits motor protein dynein, triggering lysosomal migration toward the nucleus,^16^ suggesting lysosomes may act as a highway to deliver exogenous materials into the nucleus.^15^ These lines of information support that macrophage functionality can be regulated by lysosomal pH. On the other hand, recent studies highlight that the nucleus is an innate recognition site for cGAS-STING activation.^17–19^ Currently, this pathway and the resultant type I interferon are intensively studied to improve antitumor immunotherapy.^20,21^ Therefore, we ask whether the lysosomal-nuclear machinery can be explored to fully arouse the antitumor activity of macrophages by targeting both lysosomal pH and the nuclear innate immunity.

Tumor cells can release several types of extracellular vesicles, one of which is labeled as tumor microparticles (T-MPs) with a size of 100~1000 nm.^22,23^ We have reported that T-MPs can decrease the lysosomal pH and stimulate the polarization of M1 macrophages toward an M2 phenotype,^13^ thus promoting tumor progression. However, when we used drug-loaded T-MPs to treat malignant pleural effusion (MPE) or cholangiocarcinoma in patients, a significant treatment outcome was achieved,^24,25^ hinting that drug-packaging T-MPs convert M2 TAMs in MPE to an antitumor phenotype. In addition, in our previous studies, we have found that drug-packaging MPs preferably target highly tumorigenic tumor-repopulating cells (TRCs), rather than differentiated tumor cells.^26^ This is because TRCs are softer than differentiated counterparts, leading to TRCs being more deformable and easier to take up MPs. Upon the uptake, drug-MPs enter the lysosomes and use lysosomal machinery to deliver the chemo-drugs into the nucleus to mediate TRC death.^15,26^ In addition to TRCs, drug-MPs can also be taken up by phagocytes, such as macrophages, via phagocytosis. Unlike tumor cells, which are rapidly proliferating cells, the phagocytes are likely to be terminally differentiated cells and thus are not easy to be killed by chemo-drugs.^27^ In this study, we provide evidence that chemotherapeutic drug molecules by T-MPs reset the antitumor activity of macrophages by targeting lysosomal pH and activating nuclear innate signaling. These findings highlight that drug-packaging MPs can act as a new immunotherapeutic approach by revitalizing antitumor activity of macrophages.

## Results

### Drug-MPs reset antitumor activity of macrophages in patients and mouse model

**Fig. 1:**
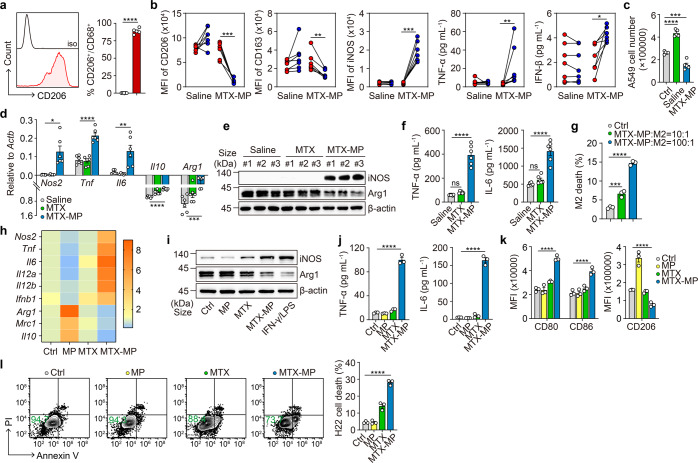
Drug-MPs reset antitumor activity of macrophages in patients and mouse model. **a** MPE from end-stage lung cancer patients was collected and the proportion of CD206^+^ macrophages within the CD68^+^ gate was detected by flow cytometry (*n* = 5). **b**, **c** MPE from end-stage lung cancer patients was collected before and after one-week treatment with intrathoracic injection of saline (*n* = 7) or MTX-MPs (*n* = 7). The expression of CD206, CD163 or iNOS within the CD45^+^CD68^+^ gate was analyzed by flow cytometry and the level of TNF-α and IFN-β in MPE was analyzed by ELISA (**b**). The CD45^+^CD68^+^ macrophages (2 × 10^6^) in MPE were isolated and incubated with 1 × 10^5^ human lung tumor A549 cells for 24 h and the number of CD45^-^ tumor cells were detected by flow cytometry, untreated A549 cells as control (*n* = 5) (**c**). **d**–**f** 1 × 10^5^ H22 tumor cells were intraperitoneally (i.p.) injected into BALB/c mice. The next day, saline, MTX (2 µg) or 2 × 10^6^ MTX-MPs (containing ~2 µg MTX) was i.p. injected into the mice every day for 15 days. A part of mice was sacrificed and *Nos2*, *Tnf*, *Il6*, *Il10* and *Arg1* expression in peritoneal macrophages was analyzed by real-time PCR (**d**), iNOS, Arg1, TNF-α and IL-6 expression was determined by western blot (**e**) and ELISA (**f**). **g** IL-4 conditioned BMDMs were treated with MTX-MPs (MPs to cells = 10:1 or 100:1) for 24 h and cell death was detected by flow cytometry. **h**–**k** IL-4 conditioned BMDMs were treated with T-MPs, MTX or MTX-MPs (MPs to cells = 10:1) for 24 h. Phenotype-associated molecular expression of viable macrophages was determined by real-time PCR (**h**), western blot (**i**), ELISA (j) and flow cytometry (**k**). **l** 2 × 10^6^ IL-4 conditioned BMDMs were treated with T-MPs, MTX or MTX-MPs and the MTX-MPs-free supernatant was collected and incubated with 1 × 10^5^ mouse H22 tumor cells for 24 h, then CD45^-^ tumor cell death was detected by flow cytometry. Unless otherwise specified, *n* = 3 biologically independent experiments were performed. Data are presented as mean ± SEM. *P* values were calculated using two-tailed unpaired Student’s *t*-tests (**a**) and paired Student’s *t*-tests (**b**) and one-way ANOVA (**c**, **d**, **f**, **g**, **j**, **k** and **l**). *P < 0.05, **P < 0.01, ***P < 0.001, ****P < 0.0001

Our previous studies used methotrexate-packaging T-MPs (MTX-MPs) to treat MPE in patients.^26,28^ We found that MTX-MPs recruit a large number of CD11b^+^CD15^+^CD49d^–^ neutrophils (78% MPE cells) to the malignant fluids, which not only display tumor cell-killing effect but also release neutrophil extracellular traps to the damaged epithelium.^28^ Apart from neutrophils, macrophages are commonly present in untreated malignant fluids.^29^ In this study, we further analyzed macrophages in the treated patients’ MPE in order to illuminate the impact of drug-MPs on the macrophages. MTX-MPs were characterized by size and drug concentration (Supplementary Fig. S1a, b), consistent with our previous reports.^26,28,30,31^ Unlike recruiting neutrophils, MTX-MPs seemed not to attract macrophages to the MPE, even lowering the percentage of CD45^+^CD68^+^ macrophages in the fluids (Supplementary Fig. S1c). We found that CD68^+^ macrophages initially expressed M2 phenotype-related CD206 (Fig. 1a), consistent with previous reports;^32,33^ following the MTX-MP treatment, however, they were converted to the M1 phenotype with the downregulation of CD206 and CD163 and the upregulation of iNOS (Fig. 1b). Notably, TNF-α and IFN-β production was increased in MPE (Fig. 1b). In line with this, the isolated macrophages from MPE of patients that had received MTX-MP treatment retarded human A549 tumor cell growth; however, macrophages from saline-treated MPE facilitated the cell growth (Fig. 1c), suggesting that MTX-MPs reverse M2 macrophages to antitumor M1 ones. H22 hepatocellular carcinoma cell line can grow quickly in the peritoneal cavity to form malignant ascites in BALB/c mice.^30^ Using this ascites model, we also demonstrated that MTX-MP treatment effectively switched macrophages in malignant ascites from M2 to M1 (Fig. 1d–f). Next, we conducted the in vitro assay by co-incubating of MTX-MPs and IL-4-conditioned M2 macrophages. Although a minority of macrophage death (<15%) was induced by the ratio of 100:1 (MPs to cells) (Fig. 1g; Supplementary Fig. S1d), we found that the cell viability was not changed (Supplementary Fig. S1d) and MTX-MPs at a ratio of either 10 or 100 could both effectively convert the viable M2 to M1, as evidenced by the upregulation of iNOS, TNF-α, IL-6, and costimulatory molecules CD80, CD86 (Fig. 1h–k; Supplementary Fig. S1e). Meanwhile, the expression of Arg1, CD206, and IL-10 was downregulated (Fig. 1h–k; Supplementary Fig. S1e). Similar results were also obtained from M0 macrophages (Supplementary Fig. S1f) or other packaged chemotherapeutic drugs, including cisplatin (Cis) (Supplementary Fig. S1g) and doxorubicin (Dox) (Supplementary Fig. S1h). Consistently, these drug-MPs treated M2 macrophages were able to cytolyze tumor cells (Fig. 1l; Supplementary Fig. S1i). Together, these findings suggest that drug-MPs can reset TAMs from an M2 phenotype to M1.

### Drug-MPs increase ROS-dependent lysosomal pH

**Fig. 2:**
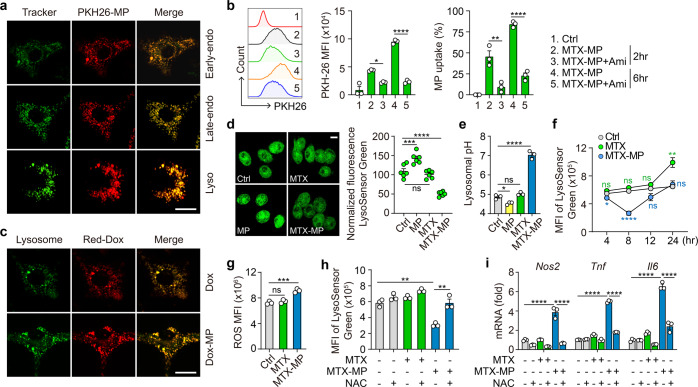
Drug-MPs increase ROS-dependent lysosomal pH. **a** IL-4 conditioned BMDMs were incubated with PKH26-labeled MTX-MPs and analyzed with early-endosomes, late-endosomes or lysosomes under a two-photon confocal microscope. **b** IL-4 conditioned BMDMs were treated with PKH26-labeled MTX-MPs in the presence or absence of amiloride hydrochloride (Ami, 75 μM) and the uptake of MTX-MPs was determined by flow cytometry. **c** IL-4 conditioned BMDMs were treated with Dox or Dox-MPs for 8 h and then the red-fluorescent Dox and lysosomes (Green) were observed under a two-photon confocal microscope. **d**, **e** IL-4 conditioned BMDMs were treated with T-MPs, MTX or MTX-MPs for 8 h. LysoSensor Green labeled acidic lysosomes were observed under a fluorescence microscope (d), pH value of lysosomes was detected by a microplate reader (**e**). **f** IL-4 conditioned BMDMs were treated with MTX or MTX-MPs and the MFI of LysoSensor Green was detected at different time points by flow cytometry. **g** IL-4 conditioned BMDMs were treated with MTX or MTX-MPs for 8 h and ROS level was detected by flow cytometry. **h-i** IL-4 conditioned BMDMs were pretreated with NAC (20 mM) for 1 h and treated with MTX or MTX-MPs. The MFI of LysoSensor was detected by flow cytometry (**h**), *Nos2*, *Tnf* and *Il6* expression was determined by real-time PCR (**i**). All scale bars, 10 μm. Unless otherwise specified, *n* = 3 biologically independent experiments were performed. Data are presented as mean ± SEM. *P* values were calculated using one-way ANOVA. *P < 0.05, **P < 0.01, ***P < 0.001, ****P < 0.0001

Next, we investigated the molecular pathway through which drug-MPs reset the antitumor activity of macrophages. Using PKH26/67-labeled MTX-MPs, we found that the MPs were initially colocalized with early endosomes, then with late endosomes, and finally with lysosomes (Fig. 2a). We did not observe the colocalization with endoplasmic reticulum, Golgi apparatus or mitochondria (Supplementary Fig. S2a). The addition of endocytotic inhibitor amiloride hydrochloride abrogated the uptake of MTX-MPs by macrophages (Fig. 2b). Doxorubicin (Dox) is a red-fluorescent agent. Using Dox-packaging T-MPs to treat macrophages, we found that Dox was also accumulated in the lysosomes (Fig. 2c), suggesting that both T-MPs and drug molecules are present in the lysosomes. Low pH is a typical feature of lysosomes. By determining lysosomal pH, we found that MTX-MPs markedly increased lysosomal pH while T-MPs alone decreased the pH (Fig. 2d, e). Dynamically, a peak of pH 7.2 was reached within 8 h and returned to the normal state 24 h later (Fig. 2f). In addition, the use of Cis-MPs or Dox-MPs to treat M2 macrophages also obtained similar results (Supplementary Fig. S2b). Studies have reported that ROS can alter lysosomal pH.^8^ An increased ROS level was found (Fig. 2g), implying a possible increase in lysosomal pH. We found that the use of ROS scavenger N-acetyl-cysteine (NAC) blocked the effect of MTX-MPs on lysosomal pH (Fig. 2h), concomitant with an M2 phenotype of macrophages (Fig. 2i). In addition, genes encoding the components of V-ATPase, the transporter which pumps proton into the lysosomes, including *V0A1*, *V0A2*, *V0A3*, *V0C*, *V0E*, *V1A*, *V1B2*, *V1C1*, *V1E1*, *V1F*, *V1G1,* and *V1H*, were not downregulated in the drug-MPs treated macrophages (Supplementary Fig. S2c). These results suggest that drug-MPs increase ROS-dependent lysosomal pH in the polarized macrophages.

### Lysosomal CYPs and NOX2 activation contribute to ROS production

**Fig. 3:**
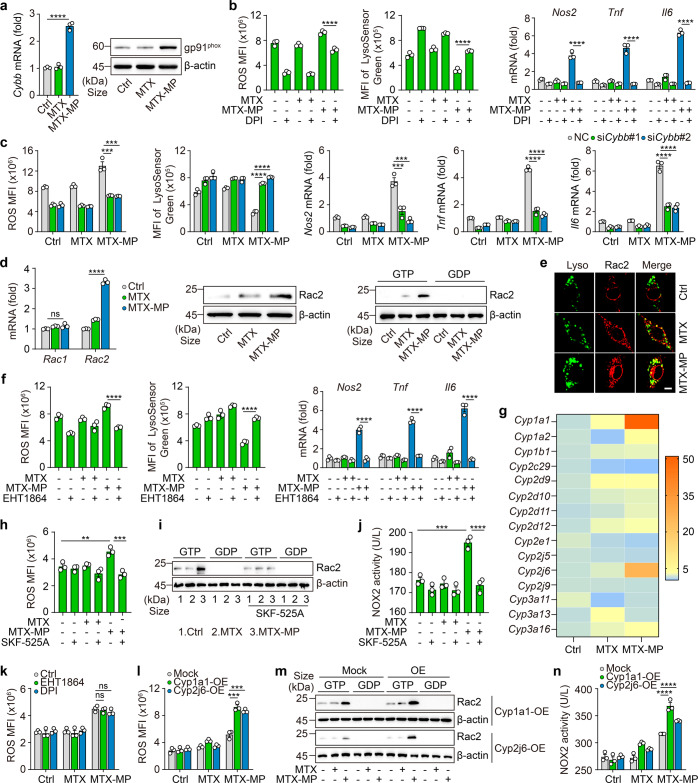
Lysosomal CYPs and NOX2 activation contribute to ROS production. **a** IL-4 conditioned BMDMs were treated with MTX or MTX-MPs for 24 h, and gp91^phox^ expression was analyzed by real-time PCR (left) and western blot (right). **b**, **c** IL-4 conditioned BMDMs were pretreated with 5 μM DPI for 30 min (**b**) or BMDMs were transfected with *Cybb* siRNAs for 12 h and stimulated with IL-4 for 12 h (**c**), then treated with MTX or MTX-MPs. ROS level and MFI of LysoSensor were detected by flow cytometry, *Nos2*, *Tnf* and *Il6* expression was determined by real-time PCR. **d**, **e** The same as (**a**), except that *Rac1* and *Rac2* expression was analyzed by real-time PCR (left), Rac2 protein level (middle) and Rac2 activity (right) were analyzed by western blot, the location of Lamp1 (green) and Rac2 (red) was observed under a two-photon confocal microscope. Scale bar, 10 μm. **f** The same as (b), except that IL-4 conditioned BMDMs were pretreated with 10 μM EHT1864 for 30 min. **g** IL-4 conditioned BMDMs were treated with MTX or MTX-MPs for 4 h, and *Cyp* subunits expression was analyzed by real-time PCR. **h**–**j** IL-4 conditioned BMDMs were pretreated with 20 μM SKF-525A for 30 min and treated with MTX or MTX-MPs for 4 h. ROS level was analyzed by flow cytometry (**h**), Rac2 activity was analyzed by western blot (**i**) and NOX2 activity was analyzed by ELISA (**j**). **k** IL-4 conditioned BMDMs were pretreated with EHT1864 or DPI for 30 min and then treated with MTX or MTX-MPs for 4 h. ROS level was detected by flow cytometry. **l**-**n** *Cyp1a1*- or *Cyp2j6*-overexpressing BMDMs were stimulated with IL-4 for 12 h and treated with MTX or MTX-MPs for 4 h, ROS level was analyzed by flow cytometry (**l**), Rac2 activity was analyzed by western blot (**m**) and NOX2 activity was analyzed by ELISA (**n**). Unless otherwise specified, *n* = 3 biologically independent experiments were performed. Data are presented as mean ± SEM. *P* values were calculated using one-way ANOVA. **P < 0.01, ***P < 0.001, ****P < 0.0001

Next, we explored the molecular basis by which drug-MPs increased the lysosomal pH. NOX2 (gp91^phox^ subunit), a multi-subunit enzyme complex, is highly used to produce lysosomal ROS by phagocytes through electron transfer from NADPH to molecular oxygen.^8^ Following the drug (MTX, Cis or Dox)-MP treatment, gp91^phox^ was upregulated in the macrophages (Fig. 3a; Supplementary Fig. S3a). Blocking NOX2 activity or expression by diphenylene iodonium (DPI) or *Cybb* siRNAs (Supplementary Fig. S3b) led to decreases in the above ROS and reduced the lysosomal pH and inflammatory cytokine expression to the levels in the untreated M2 macrophages (Fig. 3b, c). Thus, drug-MPs seem to activate the NOX2 to produce ROS in the macrophages. However, the activation of NOX2 requires an assembly of the cytosolic regulatory subunits (p47^phox^, p67^phox^, p40^phox^, and Rac) to the membrane subunits (gp91^phox^ and p22^phox^), and to achieve this, an active form of Rac is crucial.^9,34^ We found that both the expression and activity of Rac2 were increased in drug-MPs treated macrophages (Fig. 3d; Supplementary Fig. S3c, d), concomitant with its recruitment to the lysosomes (Fig. 3e). Inhibiting Rac2 activity by EHT1864 led to the decrease of ROS and lysosomal pH as well as the impaired M1 polarization (Fig. 3f). Although Rac activation is commonly mediated by classical GEFs, Rho GTPase can also be activated by ROS through reversible cysteine oxidation.^35^ Notably, disposal of chemo-drugs by lysosomal P450 monooxygenases (CYPs) can cause ROS production.^36,37^ This is because CYPs, as heme-containing enzymes, transfer NADPH-donated electrons to molecular oxygen to generate O_2_^−^ and subsequent H_2_O_2_. Notably, we found that two lysosomal CYPs (*Cyp1a1* and *Cyp2j6*) were upregulated after 4 h of drug-MP treatment (Fig. 3g; Supplementary Fig. S3e). Blocking P450 activity by SKF-525A resulted in the early decrease of ROS levels (Fig. 3h) and the inactivation of Rac2 and NOX2 (Fig. 3i, j). Moreover, neither Rac2 nor NOX2 inhibition blocked the early ROS production by drug-MPs (Fig. 3k). In addition, overexpression of Cyp1a1 or Cyp2j6 (Supplementary Fig. S3f) led to more ROS production (Fig. 3l) and further enhanced Rac2 and NOX2 activity (Fig. 3m, n). These data suggest that drug-MPs activate P450 and NOX2 for ROS production and macrophage M1 polarization.

### Lysosomal ROS and pH mediate Ca^2+^ release for macrophage M1 phenotype

**Fig. 4:**
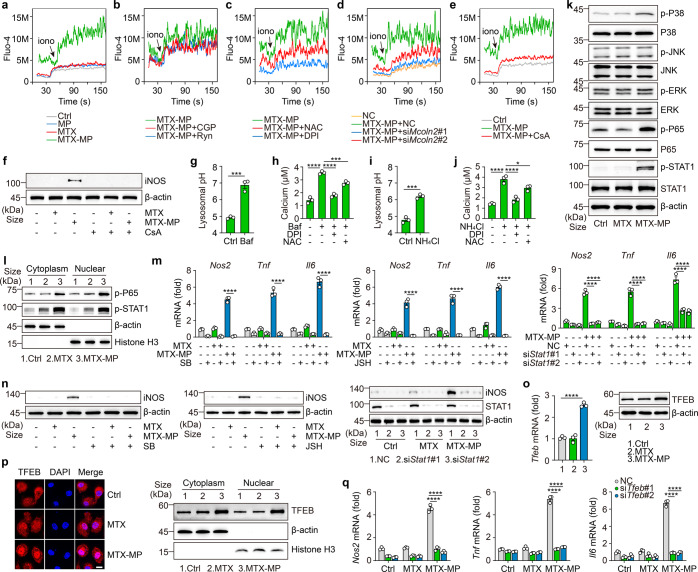
Lysosomal ROS and pH mediate Ca^2+^ release for macrophage M1 phenotype. **a** IL-4 conditioned BMDMs were treated with T-MPs, MTX or MTX-MPs for 8 h and the cytosolic calcium release was recorded by flow cytometry. **b**–**e** IL-4 conditioned BMDMs were pretreated with 100 μM ryanodine or 10 μM CGP37157 for 1 h (**b**), 20 mM NAC for 1 h, 5 μM DPI for 30 min (**c**) or 10 nM CsA for 1 h (**e**), or BMDMs were transfected with *Mcoln2* siRNAs and stimulated with IL-4 (**d**) respectively, then treated with MTX or MTX-MPs for 8 h and the cytosolic calcium release was recorded by flow cytometry. **f** IL-4 conditioned and CsA pretreated BMDMs were treated with MTX-MPs for 12 h, iNOS expression was determined by western blot. **g**, **i** IL-4 conditioned BMDMs were treated with bafilomycin A1 (**g**) or NH_4_Cl (**i**) and the pH value of lysosomes was detected by a microplate reader. **h**, **j** IL-4 conditioned and NAC or DPI-pretreated BMDMs were treated with bafilomycin A1 (**h**) or NH_4_Cl (**j**), and the cytoplasmic Ca^2+^ concentration was determined by calcium colorimetric assay. **k**, **l** IL-4 conditioned BMDMs were treated with MTX or MTX-MPs for 12 h, followed by western blot analysis of P38, JNK, ERK, P65 and STAT1. **m**, **n** IL-4 conditioned BMDMs were treated with MTX or MTX-MPs alone or combined with SB203580, JSH-23 or BMDMs were transfected with *Stat1* siRNAs for 12 h and stimulated with IL-4 for 12 h, *Nos2*, *Tnf* and *Il6* expression was determined by real-time PCR (**m**) and iNOS expression was determined by western blot (n). **o**, **p** IL-4 conditioned BMDMs were treated with MTX or MTX-MPs for 12 h, TFEB expression was analyzed by real-time PCR (**o**, left) and western blot (**o**, right), and the location of TFEB was analyzed by two-photon confocal microscope (**p**, left) and western blot (**p**, right). Scale bar, 10 μm. **q** BMDMs were transfected with *Tfeb* siRNAs for 12 h and stimulated with IL-4 for 12 h and then treated with MTX or MTX-MPs. *Nos2*, *Tnf* and *Il6* expression was determined by real-time PCR. Unless otherwise specified, *n* = 3 biologically independent experiments were performed. Data are presented as mean ± SEM. *P* values were calculated using two-tailed unpaired Student’s *t*-tests (**g**, **i**) and one-way ANOVA (**h**, **j**, **m**, **o** and **q**). *P < 0.05, ***P < 0.001, ****P < 0.0001

Next, we investigated the mechanism by which lysosome-producing ROS reset the phenotype of macrophages. Reports have indicated that Ca^2+^ signaling can activate macrophages. Given that ROS regulates lysosomal Ca^2+^ channels,^16,38^ we assumed that lysosomal ROS promoted Ca^2+^ release, thus regulating the phenotype and function of macrophages. We found that MTX-MP treatment indeed increased lysosomal Ca^2+^ (Fig. 4a), which was not affected by ryanodine or CGP37157,^39,40^ the specific inhibitor of ER or mitochondrial Ca^2+^ release (Fig. 4b). Ca^2+^ channels such as TPC1/2 and Mcoln1/2 have been reported to mediate lysosomal Ca^2+^ release.^41^ We found that *Mcoln2* expression was upregulated in the MTX-MPs treated macrophages (Supplementary Fig. S4a); however, blocking ROS by DPI or NAC resulted in the downregulation of *Mcoln2* expression (Supplementary Fig. S4b) and Ca^2+^ release (Fig. 4c). In addition, *Mcoln2* knockdown (Supplementary Fig. S4c) also decreased the above Ca^2+^ release (Fig. 4d) and the phenotype switch (Supplementary Fig. S4d). Similar results were also obtained from using Ca^2+^ signaling inhibitor cyclosporin A (CsA) (Fig. 4e, f; Supplementary Fig. S4e), suggesting that ROS production by drug-MPs contributes to lysosomal Ca^2+^ release. Given the aforementioned effect of ROS on lysosomal pH and the reported promotion of lysosomal Ca^2+^ release by increased pH,^42^ we additionally tested whether the ROS used the increased pH to promote lysosomal Ca^2+^ release. When we treated macrophages with H^+^-ATPase inhibitor bafilomycin A1 for 30 min, the lysosomal pH increased from 4.9 to 7.0 (Fig. 4g), concomitant with cytosolic Ca^2+^ increased from 1.4 to 3.6 μM (Fig. 4h). Ammonium chloride (NH_4_Cl) can rapidly increase lysosomal pH.^43^ Following the NH_4_Cl treatment for 2 min, increases in lysosomal pH and cytosolic Ca^2+^ were observed (Fig. 4i, j). Notably, the addition of DPI or NAC could not completely block bafilomycin A1- or ammonia-regulated Ca^2+^ release (Fig. 4h, j). Thus, ROS seems to directly and indirectly regulate lysosomal Ca^2+^ release. Lysosomal Ca^2+^ release leads to signal transduction. Notably, we found that mitogen-activated protein kinase p38 and nuclear factor-κB (NF-κB), two downstream molecules of Ca^2+^ signal, were activated in drug-MPs treated macrophages (Fig. 4k, l; Supplementary Fig. S4f). Also, STAT1, the key transcription factor that mediates macrophages’ proinflammatory phenotype, was activated following drug-MP treatment (Fig. 4k, l; Supplementary Fig. S4f). Using p38 or NF-κB inhibitor (SB203580 or JSH-23) or knocking down *Stat1* led to the abrogation of the effect of drug-MPs on macrophage phenotype switch (Fig. 4m, n; Supplementary Fig. S4g). Apart from these signal molecules, the lysosomal Ca^2+^ signal also regulates transcription factor EB (TFEB). We found that both TFEB expression and its nuclear localization were upregulated by drug-MP treatment (Fig. 4o, p; Supplementary Fig. S4h); and the knockdown of *Tfeb* by siRNAs (Supplementary Fig. S4i) led to the downregulation of the polarized phenotype (Fig. 4q). Together, these results suggest that increased lysosomal ROS and pH promote lysosomal Ca^2+^ release, thus triggering the phenotype switch of macrophages.

### Increased lysosomal pH facilitates transfer of lysosomal drugs to the nucleus

**Fig. 5:**
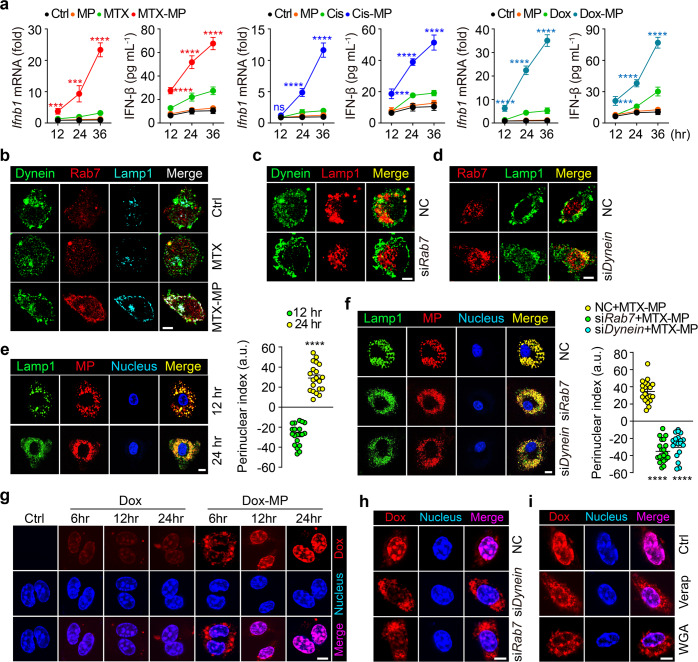
Increased lysosomal pH facilitates transfer of lysosomal drugs to the nucleus. **a** IL-4 conditioned BMDMs were treated with T-MPs, MTX/Cis/Dox or MTX-MPs/Cis-MPs/Dox-MPs for a different time, and IFN-β expression was analyzed by real-time PCR and ELISA. **b** IL-4 conditioned BMDMs were treated with MTX or MTX-MPs for 24 h and stained with anti-dynein (green), anti-Rab7 (red) and anti-Lamp1 (cyan) antibodies. **c**, **d** BMDMs were transfected with *Rab7* or *Dynein* siRNAs for 12 h and stimulated with IL-4 for 12 h, treated with MTX-MPs for 24 h, and stained with anti-Dynein (green) and anti-Lamp1 (red) antibodies or anti-Rab7 (red) and anti-Lamp1 (green) antibodies. **e** IL-4 conditioned BMDMs were treated with PKH26-labeled MTX-MPs for 12 or 24 h and stained with anti-Lamp1 (green) antibodies. DAPI was used to stain the cell nuclei (blue). The intracellular distribution of the lysosome was observed and quantified as described in the methods. **f** BMDMs were transfected with *Rab7* or *Dynein* siRNAs for 12 h and stimulated with IL-4 for 12 h, treated with PKH26-labeled MTX-MPs for 24 h and stained with anti-Lamp1 (green) antibodies. **g** IL-4 conditioned BMDMs were treated with Dox or Dox-MPs for a different time and stained with DAPI. **h** BMDMs were transfected with *Rab7* or *Dynein* siRNAs for 12 h and stimulated with IL-4 for 12 h, then treated with Dox-MPs for 24 h, and stained with DAPI. **i** IL-4 conditioned BMDMs were pretreated with Verapamil (1 mM) or WGA (75 μg mL^-1^) for 4 h and treated with Dox-MPs for 24 h, and stained with DAPI. For **b**–**i**, cells were observed under a two-photon confocal microscopy. Scale bars, 5 μm. Unless otherwise specified, *n* = 3 biologically independent experiments were performed. Data are presented as mean ± SEM. *P* values were calculated using one-way ANOVA (a and f) and two-tailed unpaired Student’s *t*-tests (e). ***P < 0.001, ****P < 0.0001

Activation of p38 and NF-κB could explain the above iNOS and TNF-α production; however, IFN-β, a cytokine that is very important in both antiviral and antitumor immunity, was also induced in drug-MPs treated macrophages (Fig. 5a). Current studies highlight the existence of DNA sensor in the nucleus which triggers type I IFN production. Given the damaging effect of some chemotherapeutic agents on DNA, we hypothesized that lysosomal chemo-drugs were delivered to the nucleus and activated the DNA sensor. Previously, we had found that T-MPs deliver both adenovirus and drugs into the nucleus of tumor cells,^26,44^ and the increased lysosomal pH may facilitate this process by recruiting Rab7 and dynein to lysosomes.^15^ In this study, we also found that Rab7 and dynein were colocalized in the lysosomal membrane in MTX-MPs treated M2 macrophages (Fig. 5b) as well as in bafilomycin A1- or ammonia-treated macrophages (Supplementary Fig. S5a). Similarly, the inhibition of lysosomal ROS by DPI or NAC did not affect bafilomycin A1- or ammonia-mediated recruitment of Rab7 and dynein (Supplementary Fig. S5a), suggesting that the increased lysosomal pH can recruit Rab7 in a ROS-independent manner. The recruitment of dynein to lysosomes could be blocked by *Rab7* knockout (Fig. 5c); however, the recruitment of Rab7 to lysosomes was not affected by *dynein* knockout (Fig. 5d), suggesting that the recruitment of dynein relies on Rab7. Meanwhile, a shorter distance between lysosomes and the nucleus was found in MTX-MPs treated macrophages (Fig. 5e). Either *Rab7* or *dynein* siRNA abrogated this shortened distance (Fig. 5f), suggesting that drug-MPs drive a centripetal movement of lysosomes. Moreover, we found that doxorubicin was gradually accumulated in the nucleus of macrophages following the Dox-MP treatment (Fig. 5g), which, however, was abrogated by *Rab7* or *dynein* siRNA (Fig. 5h). Thus, the centripetal migration seems to allow lysosomes to download drug molecules nearby the nucleus, thus facilitating drugs enter the nucleus via nuclear pores. In line with this, inhibiting drug efflux from lysosomes by verapamil or blocking nuclear pores by WGA led to the abrogation of the MP-promoted drug entry into the nucleus (Fig. 5i). In addition, using MPs packaging FITC-paclitaxel (PTX), a drug that inhibits the microtubule structures and hardly enters the nucleus to cause DNA damage, we found that the PTX was delivered nearby the nucleus (Supplementary Fig. S5b). Together, these results suggest that increased lysosomal pH facilitates the delivery of drug molecules from the lysosomes to the nucleus.

### DNA damage by drug molecules induces IFN-β via hnRNPA2B1-cGAS

**Fig. 6:**
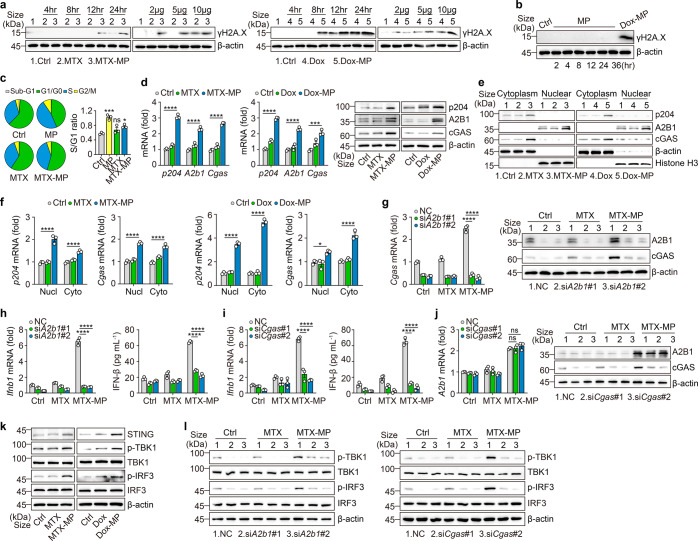
DNA damage by drug molecules induces IFN-β via hnRNPA2B1-cGAS. **a** IL-4 conditioned BMDMs were treated with different doses of MTX/Dox or MTX-MPs/Dox-MPs for a different time, and γH2A.X expression was determined by western blot. **b** IL-4 conditioned BMDMs were treated with T-MPs at different times, and γH2A.X expression was determined by western blot. **c** IL-4 conditioned BMDMs were treated with T-MPs, MTX or MTX-MPs for 24 h and the cell cycle was detected by flow cytometry. **d**, **e** IL-4 conditioned BMDMs were treated with MTX/Dox or MTX-MPs/Dox-MPs for 24 h, p204, hnRNPA2B1 and cGAS expression and location were determined by real-time PCR and western blot. **f** The same as (**d**), except that the nuclear or cytoplasmic *p204* and *Cgas* mRNAs were detected by real-time PCR. **g**, **h** BMDMs were transfected with *hnRNPA2B1* siRNAs for 12 h and stimulated with IL-4 for 12 h, and treated with MTX or MTX-MPs for 24 h, cGAS and hnRNPA2B1 expression was determined by real-time PCR and western blot (**g**). IFN-β expression was determined by real-time PCR and ELISA (**h**). **i**, **j** the same as (**g**, **h**), except that IL-4 conditioned BMDMs were transfected with *Cgas* siRNAs. **k** IL-4 conditioned BMDMs were treated with MTX or MTX-MPs for 24 h, followed by western blot analysis of STING, TBK1, and IRF3. **l** BMDMs were transfected with *hnRNPA2B1* or *Cgas* siRNAs for 12 h and stimulated with IL-4 for 12 h, and treated with MTX or MTX-MPs for 24 h, followed by western blot analysis of TBK1 and IRF3. Unless otherwise specified, *n* = 3 biologically independent experiments were performed. Data are presented as mean ± SEM. *P* values were calculated using one-way ANOVA. ***P < 0.001, ****P < 0.0001

Nuclear DNA damage by chemotherapeutic drugs can be marked by the Ser139- phosphorylated H2A.X (γH2A.X).^45^ We found that drug (MTX, Dox or Cis)-MPs treated macrophages promoted H2A.X phosphorylation in a dose- and time-dependent manner (Fig. 6a; Supplementary Fig. S6a). However, T-MPs or T-MPs packaging PTX did not cause H2A.X phosphorylation (Fig. 6b; Supplementary Fig. S6b). On the other hand, we found that although replication stalling was not induced, T-MPs could increase the ratio of the S phase and G1/G0 phase of M2 macrophages (Fig. 6c). To date, several nuclear DNA sensors have been identified, including IFN-γ-inducible protein 16 (IFI16), heterogeneous nuclear ribonucleoprotein A2B1 (hnRNPA2B1) and nuclear cGAS.^17,18^ Upon MTX-MP or Dox-MP treatment, these DNA sensors were upregulated in the M2 macrophages (Fig. 6d); however, only hnRNPA2B1 but not p204 (the functional mouse ortholog of human IFI16) or cGAS was accumulated in the nucleus (Fig. 6e). Similar results were obtained from Cis-MPs treated M2 macrophages (Supplementary Fig. S6c). Notably, hnRNPA2B1 has been reported to participate in mRNA nuclear export, cytoplasmic stability and translation.^17^ In line with this, we found that both the nuclear expression and cytoplasmic translocation of *p204* and *Cgas* mRNAs were increased (Fig. 6f; Supplementary Fig. S6d), and *hnRNPA2B1* knockout markedly decreased cGAS at both mRNA and protein levels (Fig. 6g). Also, knocking out *hnRNPA2B1* disrupted the induction of IFN-β in the treated M2 macrophages (Fig. 6h). In addition, *cGAS* knockout, although disrupting the effect of drug-MPs on IFN-β (Fig. 6i), had a minor effect on hnRNPA2B1 (Fig. 6j). Thus, MP-delivered chemo-drugs in the nucleus activated hnRNPA2B1 for IFN-β production. In line with this, drug-MP treatment resulted in the phosphorylation of TBK1 and IRF3, the downstream molecules of cGAS-STING signaling in the macrophages (Fig. 6k; Supplementary Fig. S6e), which, however, was abrogated by the knockout of *hnRNPA2B1* or *cGAS* (Fig. 6l). Together, these results suggest that drug molecules by T-MPs enter the nucleus and trigger the hnRNPA2B1-cGAS signaling pathway for IFN-β production.

### Drug-MPs synergize PD-1 blockade to treat malignant ascites

**Fig. 7:**
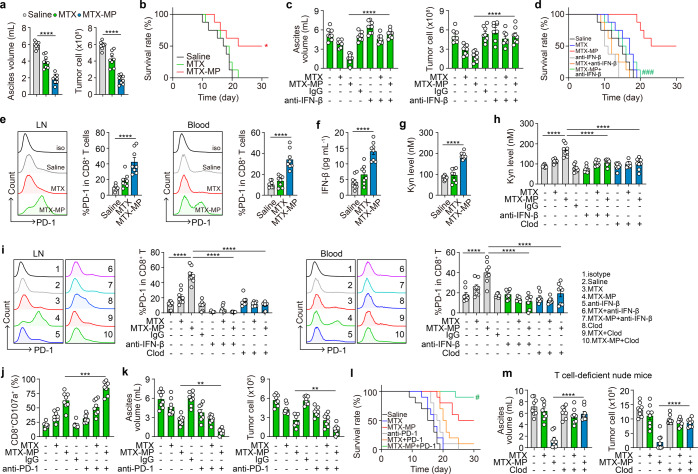
Drug-MPs synergize PD-1 blockade to treat malignant ascites. **a**, **b** 1 × 10^5^ H22 tumor cells were intraperitoneally (i.p.) injected into BALB/c mice. The next day, saline, MTX (2 µg) or 2 × 10^6^ MTX-MPs (containing ~2 µg MTX) were i.p. injected into the mice once every day for 15 days. On day 15, mice were sacrificed. The ascites volume and number of CD45^-^ tumor cells were determined (**a**). The long-term survival of tumor-bearing mice was assessed (*n* = 8) (**b**). **c, d** the same as (**a**, **b**), except that some mice were i.p. injected with IgG or anti-IFN-β neutralizing antibodies (100 µg/mouse) once per 2 days for 15 days (*n* = 8). #, MTX-MPs vs MTX-MPs+anti-IFN-β group. **e**–**g** the same as (**a**), except that CD8^+^PD-1^+^ cells of mesenteric lymph node (LN) and peripheral blood were analyzed by flow cytometry (**e**), IFN-β (**f**) and Kyn level (**g**) in ascites were analyzed by ELISA. **h**, **i** the same as (**c**), except that some mice were i.p. injected with clodronate liposomes and Kyn level (**h**) in ascites was analyzed by ELISA, CD8^+^PD-1^+^ cells of mesenteric lymph node (LN) and peripheral blood were analyzed by flow cytometry (**i**). **j**–**l** the same treatment as (a), except that some mice were i.p. injected with IgG or anti- PD-1 neutralizing antibodies (250 µg/mouse) once per 2 days for 15 days. CD8^+^CD107a^+^ cells were analyzed by flow cytometry (n = 8) (**j**), ascites volume and number of CD45^-^ tumor cells were determined (*n* = 8) (**k**) and the long-time survival of mice was assessed (*n* = 10). #, MTX-MPs vs MTX-MPs+anti-PD-1 group (**l**). **m** 1 × 10^5^ H22 tumor cells were i.p. injected into nude mice. The next day, saline, MTX or MTX-MPs were i.p. injected into the mice once every day for 15 days. At the same time, some mice were i.p. injected with clodronate liposomes for 15 days. On day 15, mice were sacrificed. The ascites volume and number of CD45^-^ tumor cells were determined. Unless otherwise specified, *n* = 3 biologically independent experiments were performed. Data are presented as mean ± SEM. *P* values were calculated using one-way ANOVA (**a**, **c**, **e**–**k** and **m**) and a two-sided log-rank (Mantel-Cox) test (**b**, **d** and **l**). *P < 0.05, **P < 0.01, ***P < 0.001, ****P < 0.0001, ^#^*P* < 0.05, ^###^*P* < 0.001

The elucidation of the above molecular basis enabled drug-MPs as a candidate to treat malignant ascites, common clinically intractable metastatic lesions, considering (1) malignant ascites is a rich source of TAMs; (2) malignant ascites occurs frequently but its management remains palliative; and (3) drug-MPs can be easily delivered into ascites via a catheter. First, we found that in the H22 ascites model, MTX-MP treatment decreased ascites volume and tumor cell number (Fig. 7a) and prolonged the survival of the mice (Fig. 7b). Similar results were also obtained from cis-MPs and Dox-MPs (Supplementary Fig. S7a, b). Moreover, we found that neutralizing IFN-β markedly promoted tumor growth and ascites formation (Fig. 7c) and shortened the survival of the mice (Fig. 7d), suggesting that IFN-β plays a critical role in mediating the antitumor effect of drug-MPs against malignant ascites. However, this single treatment was likely to promote PD-1 expression and thus dampen its efficacy, considering that (1) M1 macrophage-released factors may boost CD8^+^ T cell activation, which enhances the induction of PD-1 expression;^46^ and (2) IFN-β can stimulate tumor cells to release kynurenine (Kyn) and the latter induces PD-1 upregulation by CD8^+^ T cells via AhR activation.^47^ Indeed, we found that upon MTX-MP treatment, PD-1 expression was upregulated in both mesenteric lymph node and peripheral blood CD8^+^ T cells (Fig. 7e). In line with this, increased levels of IFN-β and Kyn in the ascites were found (Fig. 7f, g), and IFN-β neutralization or macrophage depletion reduced Kyn levels in the ascites (Fig. 7h) and downregulated the expression of PD-1 in CD8^+^ T cells (Fig. 7i). These results rationalized drug-MPs and PD-1 antibody as combined immunotherapy against malignant ascites. Upon MTX-MP treatment, ascites macrophages and CD8^+^ T cells exhibited the ability to cytolyze H22 tumor cells in vitro; and the combined PD-1 Ab treatment further enhanced this outcome (Supplementary Fig. S7c, d), concomitant with the increased expression of CD107a (Fig. 7j). In line with this, the combination of MTX-MPs and anti-PD-1 reduced ascites to a less level (Fig. 7k) and permitted a longer survival of the mice (Fig. 7l), compared to single MTX-MP treatment. In addition, the treatment efficacy of MTX-MPs was also observed in T cell-deficient nude mice, which, however, was abrogated by macrophage depletion (Fig. 7m). In addition, using the solid tumor model (B16 melanoma), we also demonstrated the conversion of M2 TAMs to M1 by MTX-MP treatment and the efficacy of the combined MTX-MPs and PD-1 Abs against B16 melanoma (Supplementary Fig. S7e-g). Together, these results suggest that drug-MPs activate both peritoneal macrophages and tumor-reactive T cells and can synergize PD-1 blockade for malignant ascites treatment.

## Discussion

**Fig. 8:**
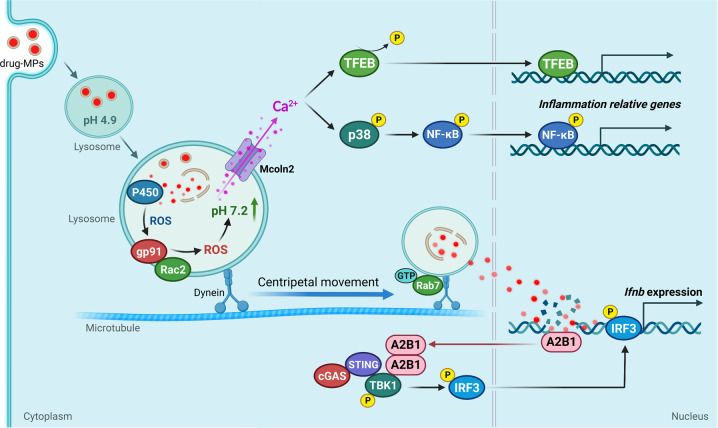
The schematic diagram of drug-MPs resetting antitumor activity of macrophages. Drug-MPs increase TAMs lysosomal pH by activating lysosomal P450 monooxygenases and NOX2, causing Ca^2+^ release via the lysosomal Ca^2+^ channel mucolipin 2 (Mcoln2), which induces the activation of p38, NF-κB, and TFEB, thus polarizing TAMs to produce inflammatory cytokines. Meanwhile, drug molecules are delivered by the lysosomes into the nucleus, where they activate DNA sensor hnRNPA2B1 for IFN-β production. This figure created with BioRender.com

Despite that extracellular vesicles are ideal to be used as drug carriers,^24,26,28^ how to use these natural biomaterials for cancer immunotherapy remains largely to be explored.^48,49^ In this study, we provide evidence that drug-MPs reset M2 macrophages to M1 by activating lysosomal cytochrome P450 and nuclear hnRNPA2B1 (Fig. 8), thus facilitating the neutrophil recruitment^24,28^ and enhancement of antitumor immunity.

It has been known that tumor MPs rather than normal cell-derived MPs can polarize TAMs to M2 for tumor progression.^50^ In addition, chemotherapeutic drug treatment also induces M2 polarization. Enigmatically, putting tumor MPs and drug molecules together results in the switch of TAMs from M2 to M1. The secret is the lysosome. Lysosomes physiologically have a low pH around 4.5–5.0. On one hand, different enzymes in lysosomes continually degrade materials at the expense of consuming protons; on the other hand, lysosomal vacuolar-type H^+^-ATPase (V-ATPase), composing of V0 and V1 domains, continually pumps protons from the cytosol into the lysosomal lumen in order to replenish the proton loss.^7^ Of note, MPs and MTX-MPs may have different effects on lysosomal pH. MPs decrease but drug-MPs increase the lysosomal pH of macrophages, as shown in Fig. 2e, f. Although the mechanism underlying MP-mediated decrease of lysosomal pH has not been fully elucidated, the oxidized cholesterol in tumor cell-derived MPs might be involved in the mechanism. Such oxidization is mediated by the cholesterol 25-hydroxylase, which is upregulated in the irradiated tumor cells during the preparation of tumor cell-derived MPs. Knocking out the enzyme and using the MPs without oxidized cholesterol to repeat the experiment, the result showed that lysosomal pH did not decrease.^51^ In addition, the use of normal cell-derived MPs and liposomes, both of which do not contain oxidized cholesterol, to treat macrophages also did not alter the lysosomal pH.^51^ Thus, it is possible that the oxidized cholesterol from tumor MPs may generate signals, which regulate V-ATPase. As a support, we found that MPs are able to upregulate V-ATPase subunits V0a2 and V0a3.^13^ However, drug-MPs does not alter the expression of V-ATPase subunits. Notwithstanding this, once entering lysosomes, drug molecules can be catalyzed by lysosomal P450 monooxygenases, leading to the generation of superoxide anion, which then triggers the activation of lysosomal NOX2 system to amplify superoxide anion. In the lysosomal lumen, superoxide is quickly converted into hydrogen peroxide by consuming protons, leading to the increase of lysosomal pH. P450 monooxygenases evolve to detoxify xenobiotics, which are mainly located on ER membrane,^52^ which, however, can also be located in mitochondria, plasma membrane and lysosomes.^10,53,54^ Thus, lysosomal P450 enzymes can catalyze chemo-drug molecules by adding a single oxygen atom, concomitant with the generating superoxide anion. Unlike conventional chemo treatment, which allows few drug molecules in lysosomes, the drug-MP treatment delivers numerous drug molecules to the lysosomal lumen, thus generating enough ROS to activate the lysosomal NOX2 system and the subsequent ROS amplification.

IFN-γ or LPS-stimulated macrophages with M1 phenotype usually do not express IFN-β, the important cytokine required for CD8^+^ T cell priming. The cGAS-STING pathway is crucial for IFN-β production by activating IRF3/7.^55,56^ In addition to various cGAS-STING agonists, physical irradiation is also explored for tumor immunotherapy by inducing the cGAS-STING activation.^57^ In this study, we found that chemotherapeutic drugs can act as cGAS-STING agonists through the lysosomal-nuclear pathway. Tumor chemo-drugs delivered by tumor MPs effectively activate the P450-NOX2 pathway, leading to abundant ROS production in lysosomes of macrophages. Such ROS consumes protons and causes increases in lysosomal pH. As a result, increased lysosomal pH recruits motor protein dynein to the lysosomal membrane via the small GTPase Rab7, thus mediating lysosomal centripetal movement along microtubule tracks towards the nucleus, where drug molecules are downloaded and enter the nucleus via nuclear pores. These nucleus-accumulated chemo-drug molecules can cause DNA damage, such as dsDNA breaks. Recent studies have found that nucleus-localized DNA sensors, such as IFN-γ-inducible protein 16 (IFI16), heterogeneous nuclear ribonucleoprotein A2B1 (hnRNPA2B1) and nuclear cGAS, can recognize damaged-DNA, leading to the activation of the cGAS-STING-TBK1-IRF3 signaling cascade and the transcriptional activation of IFN-β.^17–19^ Such generated IFN-β may play a crucial role in the resolution of malignant fluids, since IFN-β neutralization markedly impairs the inhibitory effect of MTX-MPs on H22 ascites (Fig. 7c, d). This might reveal an important property of IFN-β in the anti-ascites aspect. Malignant ascites is caused by tumor cell growth in the peritoneal cavity, but the direct reason is the increased capillary permeability of peritoneum. IFN-β is well known for its anti-angiogenic effect,^58^ which may reduce the permeability and inhibit the ascites formation. Although IFN-β is pivotal during T cell priming, using T cell-deficient nude mice we still can observe certain treatment efficacy of drug-MPs (Fig. 7m), suggesting that drug-MPs may mobilize other immune factors beyond T cells to exert the anti-ascites effect; however, such effect can be abrogated by macrophage depletion, suggesting drug-MPs mobilize macrophages for its treatment efficacy. In addition, these treatment outcomes do not exclude the effects of drug-MPs on neutrophils or tumor cells. In fact, our previous studies have demonstrated that drug-MPs can effectively kill tumor cells especially for highly tumorigenic cells and can attract and activate neutrophils to exert the antitumor effects.^24,26,28,30^

MPE poses a significant clinical problem with a poor treatment efficacy.^59^ We previously reported that drug-MPs could be a useful approach to tackle patients’ MPE.^28^ In this study, we further tested the efficacy in the treatment of malignant ascites, an even more complex malignant fluid relative to MPE, in the animal model by the combination with PD-1 blockade. Abundant macrophages normally reside in the peritoneal cavity, and once the formation of malignant fluid, more macrophages are accumulated in the cavity, which exhibits an M2 phenotype.^60^ Thus, macrophages might become an innate immune checkpoint in malignant ascites. The combination of drug-MPs and PD-1 antibodies provides a rationale for tackling malignant ascites by relieving both innate and adaptive immune checkpoints. Indeed, this combination shows the treatment efficacy in the animal model, which guarantees its trial in the clinic. In fact, targeting both TAMs and PD-1/PD-L1 is currently highlighted as a combined immunotherapeutic approach.^61,62^ In addition, considering surgery-induced immunosuppression and TAMs in the wound after surgery, the post-surgery local administration of drug-MPs might ameliorate the immunosuppressive state of the wound, which may favor the control of the tumor relapse. All in all, we provide evidence that drug-MPs functions as an immune modulator and mediates its antitumor efficaciousness by resetting TAMs to an antitumor phenotype.

## Materials and methods

### Human Samples

Malignant pleural effusion in patients with non-small cell lung cancer (NSCLC) was collected from Union Hospital, affiliated with Tongji Medical College of Huazhong University of Science and Technology. The patient’s treatment schedule and MPE collection were described as previously.^28^ The Clinical Trials Ethics Committee of the Huazhong University of Science and Technology approved the clinical trial [NO: (2015) 0702-2]. All patients signed informed consent forms to participate in the study.

### Animals and Cell lines

Female wild-type C57BL/6 J, BALB/c mice and nude mice (5-8-week) were purchased from Vital River Laboratory Animal Technology Co. Ltd (Beijing, China). For this study, all mice were housed in an SPF animal facility of Tongji Medical College Animals. All experimental procedures were approved by the Animal Care and Use Committee of Tongji Medical College. Human lung carcinoma A549, murine melanoma B16 and hepatocarcinoma H22 cell lines were obtained from China Center for Type Culture Collection (Wuhan, China) and cultured in DMEM or RPMI1640 medium (Thermo Scientific) with 10% FBS (Gibco) at 37 °C with 5% CO_2_.

### Reagents

Doxorubicin (Dox, MB1087-1), Methotrexate (MTX, MB1156-1), Cisplatin (Cis, MB1055-1) and Paclitaxel (PTX, MB1178-1) were purchased from Meilunbio. Amiloride hydrochloride (PHR1839), NAC (A9165), DPI (43088), cyclosporin A (CsA, SML1018), EHT1864 (E1657), SKF-525A (567300), SB203580 (S8307), JSH-23 (J4455), PKH26-Red fluorescent cell linker kit, NH_4_Cl (A9434), PKH67-Green fluorescent cell linker kit and anti-Lamp1 antibody (L1418) were purchased from Sigma-Aldrich. Ryanodine (ab120083), CGP37157 (ab120012) were purchased from Abcam. Bafilomycin A1 (HY-100558) were purchased from MCE. ER-, Mito-, Lyso-Tracker Green/Red, LysoSensor Yellow/Blue DND-160 (L7545), Early Endosomes-GFP (C10586), LysoSensor Green DND-189 (L7535), CellLight Golgi-RFP (C10593) and Late Endosomes-GFP (C10588) were purchased from Thermo Fisher Scientific. Clodronate liposomes were purchased from Liposomal.

### Preparation of macrophages

Primary BMDMs were generated by culturing mouse bone marrow cells in the presence of 20 ng mL^−1^ M-CSF (315-02, PeproTech) conditional medium for 5 days. On day 6, BMDMs were stimulated with 100 ng mL^−1^ LPS plus 20 ng mL^−1^ IFN-γ (315-05, PeproTech) or 10 ng mL^−1^ IL-4 (214-14, PeproTech) for 24 h, producing M1 or M2 macrophages respectively. For isolation of peritoneal macrophages, peritoneal lavage was performed with cold PBS, the cells were collected and seeded on a six-well plate. The adhesion cells were collected as peritoneal macrophages (CD45^+^F4/80^+^å 90%). For isolating human monocytes/macrophages from MPE, the MPE was centrifuged and the cells were purified with human CD14 Micro-Beads (130-050-201, MACS).

### Preparation of drug-packaging MPs

2.5 × 10^8^ A549 cells or 3 × 10^8^ H22 cells in 20 mL culture media were exposed to ultraviolet irradiation (UVB, 300 J m^−2^) for 1 h. Then, 1 mg mL^−1^ MTX, 100 μg mL^−1^ Cis or 500 μg mL^−1^ Dox was added to the culture medium. 16 h later, supernatants were collected and the centrifugation method has been described previously.^26,28^ The drug-packaging MPs were quantified and characterized using a nanoparticle tracking analysis (NTA) system (Nanosight NS300, Malvern) as described previously.^28^ The drug concentration was detected by high-performance liquid chromatography (HPLC) and analyzed as described previously.^26,28^ For the clinical trial, the process was conducted under good manufacturing practice (GMP) quality standards.

### Real-time PCR

Total RNA was prepared and treated with Trizol (15596026, Invitrogen), according to the manufacturer’s protocol. Reverse transcription was performed using the ReverTra Ace qPCR RT Kit (FSQ-101, Toyobo), and real-time PCR was performed. The primer sequences are shown in Supplementary Table S1.

### Plasmids and transfection

*Cyp1a1* and *Cyp2j6* plasmids were purchased from YouBio (Hunan, China) and transiently transfected into BMDMs with Lipofectamine RNAiMAX Transfection Reagent (13778150, Invitrogen). Sequencing primers: *Cyp1a1* forward, 5′-CATCACAGACAGCCTCATTGA GC-3′ and reverse, 5′-CTCCACGAGATAGCAGTTGTGAC-3′, *Cyp2j6* forward, 5′-GGACCTCTTCTTTGCTGGAACAG-3′ and reverse, 5′-GCAAGTCTTGCTGCCCTCT TCT-3′.

### siRNA transfection

Macrophages were transfected with 50 nM siRNAs (RiboBio, Guangzhou, China), utilizing Lipofectamine RNAiMAX according to the manufacturer’s protocol. The siRNA sequences are shown in Supplementary Table S2.

### Western blot analysis and ELISA

Cultured cells were homogenized in NP40 (Beyotime), and equal amounts of lysates were separated on 10%/12% SDS-PAGE and transferred onto nitrocellulose membranes. After blocking with 5% fat-free milk, the membranes were probed with specific antibodies overnight at 4 °C and then washed and incubated with HRP conjugated antibodies. Proteins were visualized with chemiluminescence (ECL Kit, 34577, Pierce). The primary antibodies are shown in Supplementary Table S3. Mouse IFN-β (439407, Biolegend), TNF-α (430904, Biolegend) and IL-6 (431307, Biolegend), human IFN-β (lurex-hifnbv2, InvivoGen) and TNF-α (BGK01375, PeproTech) production in the supernatants was assessed by ELISA kits according to the manufacturer’s instructions.

### Flow cytometric analysis

Macrophages were stained with surface antibodies according to the manufacturer’s guidelines. For the detection of intracellular proteins, the cells were fixed, then ruptured the membrane with permeabilization buffer and stained with antibodies. The antibodies are shown in Supplementary Table S4. For CD45 cell isolation, total cells were collected from MPE, washed with PBS and purified CD45 cells using human or mouse CD45 Micro-Beads (#130-045-801, MACS). Data were acquired on an Accuri C6 (BD Biosciences) and analyzed with FlowJo software. Mouse M2 phenotype: CD45^+^F4/80^+^CD163^+^/CD206^+^, Mouse M1 phenotype: CD45^+^F4/80^+^iNOS^+^/CD80^+^/CD86^+^. Human M2 phenotype: CD45^+^CD68^+^CD163^+^/CD206^+^; Human M1 phenotype: CD45^+^CD68^+^iNOS^+^.

### Lysosomal pH value assay

The lysosomal pH value: the cells were collected and then stained with 10 μM LysoSensor Yellow/Blue for 5 min at 37 °C in 1640 medium and washed with PBS. Then the cells were transferred into a black 96-well plate and treated with 10 μM monensin and 10 μM nigericin in the calibration buffer (pH 4.5–7.5). Fluorescence was measured on Ex-360/Em-440 and Ex-360/Em-550 using a microplate reader (Synergy H1, BioTek). For LysoSensor Green probes: the cells (3 × 10^6^ cells per mL) were incubated with probes for 30 min at 37 °C and then washed with PBS, immediately analyzed by fluorescence microscope or flow cytometry.

### Detection of reactive oxygen species

The cells were collected and then washed with PBS, pelleted at 400 × *g* for 5 min, re-suspended in PBS containing 500 nM CellROX Green (C10444, Invitrogen) for 30 min at 37 °C and analyzed by flow cytometry at 488-nm excitation.

### Intracellular Ca^2+^ measurement

Intracellular Ca^2+^ measurement has been described previously.^13,16^ For intracellular calcium concentration assay: the cells were collected and then treated with 100 μL sample lysate. After complete lysis, centrifuged at 14,000 × *g* for 5 min, and the supernatants were quantified by Calcium Colorimetric Assay Kit (S1063S, Beyotime).

### Detection of Rac2 and NOX2 activity

The Rac2 activity was measured by the Rac2 Activation Assay kit (ab211162, Abcam). The NOX2 activity was measured by NADPH oxidase 2 Assay kit (H327-1, Nanjing Jiancheng). Measurements were performed according to the manufacturer’s instructions.

### Quantification of lysosome distribution

Lysosome distribution was quantified as described previously.^63^ Briefly, the number of lysosomes in the whole cell (*I* total), in the area within 10 μm of the nucleus (*I* perinuclear), and the area >15 μm from the nucleus (*I* peripheral) were counted. The perinuclear and peripheral normalized intensities were first calculated and normalized as *I*_<10_ = *I* perinuclear/*I* total and *I*_>15_ = *I* peripheral/*I* total. The perinuclear index was defined as (*I*_<10_ -*I*_>15_) ×100. 20 cells’ perinuclear index was quantified in every group. Researchers did quantifications blindingly to the experimental groups presented.

### Data statistical analyses

All experiments were performed at least three times. Results were expressed as mean ± SEM and analyzed by a two-tailed unpaired Student *t-*test or one-way ANOVA. In all tests, *p* values of <0.05 were considered statistically significant. The analysis was conducted using the GraphPad Prism 8.0 software.

## Supplementary information

Supplemental information

Supplemental information

## Data Availability

The data used in the study are available from the corresponding authors upon reasonable request.
