# Supplementary material for: Chemo-drugs in cell microparticles reset antitumor activity of macrophages by activating lysosomal P450 and nuclear hnRNPA2B1
Source: Signal Transduct Target Ther. 2023 Jan 20;8:22. doi: 10.1038/s41392-022-01212-7 (PMC9852455; doi:10.1038/s41392-022-01212-7)
Supplement: Supplementary file 1 — Supplemental information [file 41392_2022_1212_MOESM1_ESM.doc]

Supplementary Materials for

**Chemo-drugs in cell microparticles reset antitumor activity of macrophages by activating lysosomal P450 and** **nuclear hnRNPA2B1**

Keke Wei, Huafeng Zhang, Shuaishuai Yang, Yuxiao Cui, Bingxia Zhang, Jincheng Liu, Liang Tang, Yaoyao Tan, Simin Liu, Shiqi Chen, Wu Yuan, Xiao Luo, Chen Chen, Fei Li, Junwei Liu, Jie Chen, Pingwei Xu, Jiadi Lv, Ke Tang, Yi Zhang, Jingwei Ma*, Bo Huang*

*Correspondence to: Bo Huang (tjhuangbo@hotmail.com) and Jingwei Ma (majingwei@hust.edu.cn)

**This PDF file includes:**

Figures. S1 to S7

Tables S1 to S4





**Fig. S1** **Drug-MPs reset antitumor activity of macrophages in patients and mouse model. a** Size distribution of H22 tumor cells derived MTX-MPs was measured by nanoparticle tracking analysis (NTA) system. **b** 3×10^8^ H22 tumor cells derived MTX-MPs were prepared and the loaded MTX was measured by high performance liquid chromatography (HPLC). MTX was quantified by the Ultimate 3000 HPLC system on a C^18^ column, and MTX concentration was calculated according to the standard curve. **c** MPE from end-stage lung cancer patients was collected before and after one-week treatment with intrathoracic injection of saline (*n*=7) or MTX-MPs (*n*=7). The population of CD45^+^CD68^+^ macrophages (Mac) in MPE cells was analyzed by flow cytometry. **d** Unstimulated BMDMs (M0) were treated with MTX-MPs (MPs to cells=10:1 or 100:1) for 24 hours and cell death was detected by flow cytometry (d, left). IL-4 conditioned BMDMs were treated with MTX or MTX-MPs for 24 hours and cell viability was detected by CCK8 analysis (d, right). **e** IL-4 conditioned BMDMs were treated with T-MPs, MTX or MTX-MPs (MPs to cells=100:1) for 24 hours and phenotype-associated molecular expression of viable macrophages was determined by real-time PCR. **f-h** Unstimulated BMDMs (M0) or IL-4 conditioned BMDMs were treated with MTX/Cis/Dox or MTX-MPs/Cis-MPs/Dox-MPs respectively for 24 hours. Phenotype-associated molecular expression of viable macrophages was determined by real-time PCR, western blot, ELISA and flow cytometry. **i** 2×10^6^ IL-4 conditioned BMDMs were treated with Cis or Cis-MPs and the Cis-MPs-free supernatant was collected and incubated with 1×10^5^ mouse H22 tumor cells for 24 hours, then CD45^-^ tumor cell death was detected by flow cytometry. Unless otherwise specified, n=3 biologically independent experiments were performed. Data are presented as mean ± SEM. *P* values were calculated using one-way ANOVA. **P*<0.05, ***P*<0.01, ****P*<0.001, *****P*<0.0001.


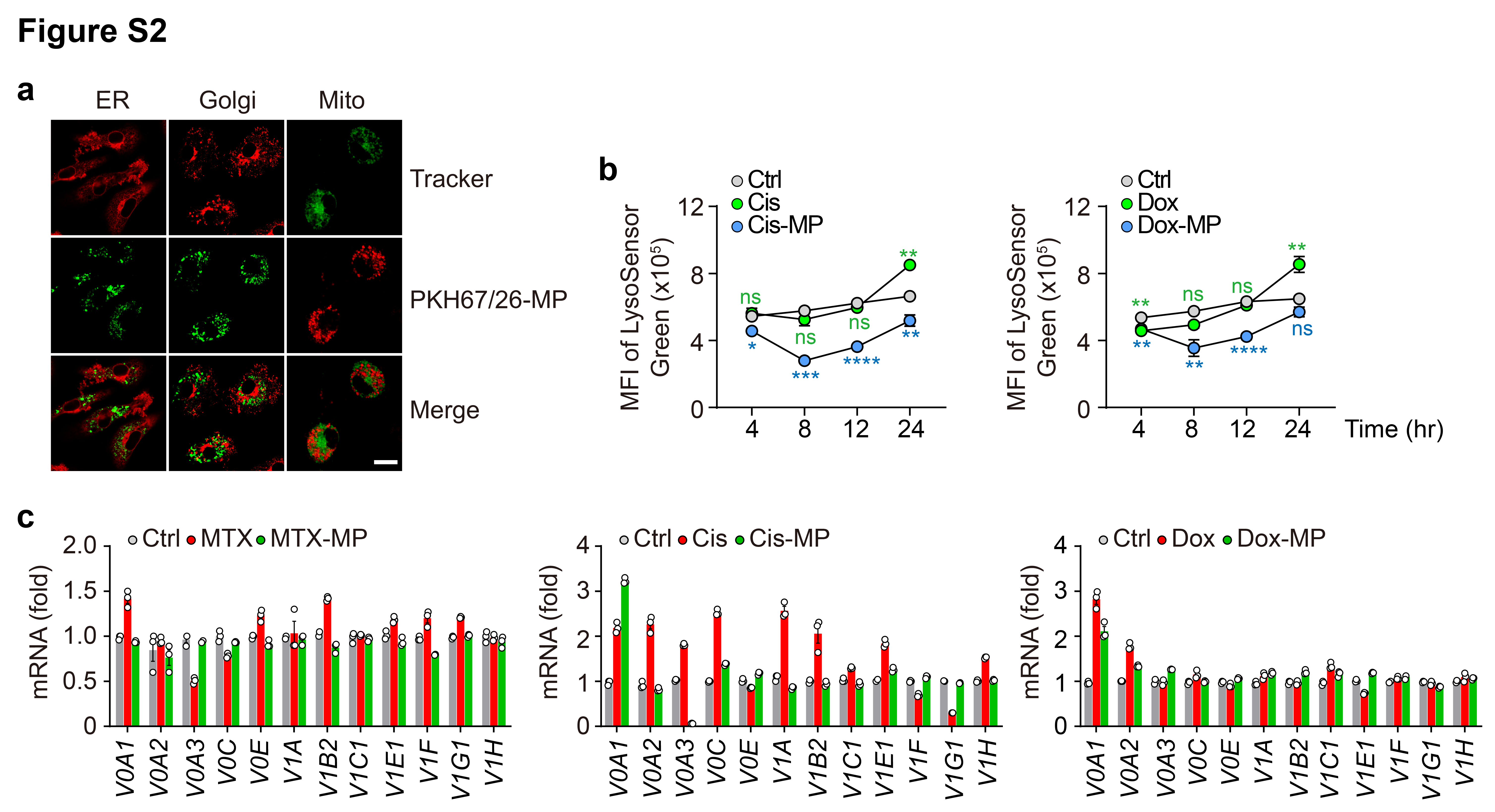


**Fig. S2.** **Drug-MPs increase ROS-dependent lysosomal pH.** **a** IL-4 conditioned BMDMs were incubated with PKH26/67-labeled MTX-MPs and analyzed with ER, Golgi or mitochondria Red/Green Trackers under a two-photon confocal microscope. Scale bars, 10 μm. **b** IL-4 conditioned BMDMs were treated with Cis/Dox or Cis-MPs/Dox-MPs and the MFI of LysoSensor Green was detected at different time points by flow cytometry. **c** IL-4 conditioned BMDMs were treated with MTX/Cis/Dox or MTX-MPs/Cis-MPs/Dox-MPs for 8 hours and *V-ATPase* subunits expression was analyzed by real-time PCR. Unless otherwise specified, n=3 biologically independent experiments were performed. Data are presented as mean ± SEM. *P* values were calculated using one-way ANOVA. **P*<0.05, ***P*<0.01, ****P*<0.001, *****P*<0.0001.


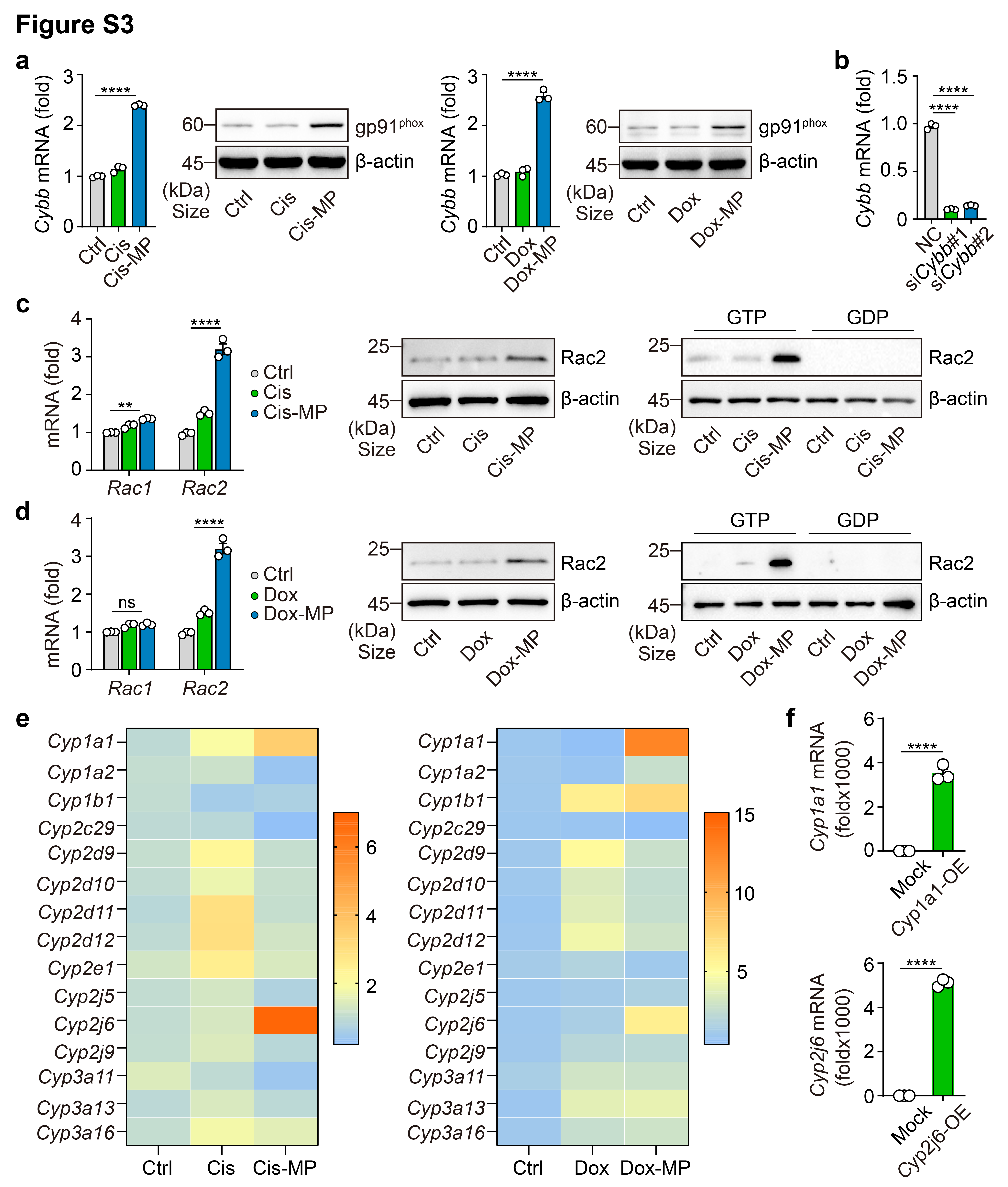


**Fig. S3.** **Lysosomal CYPs and NOX2 activation contribute to ROS production. a** IL-4 conditioned BMDMs were treated with Cis/Dox or Cis-MPs/Dox-MPs for 24 hours and gp91^phox^ expression was analyzed by real-time PCR and western blot. **b** BMDMs were transfected with *Cybb* siRNAs for 12 hours and stimulated with IL-4 for 12 hours, *Cybb* expression was determined by real-time PCR. **c-d** the same as (a), except that *Rac1* and *Rac2* expression was analyzed by real-time PCR (left), Rac2 protein level (middle) and Rac2 activity (right) were analyzed by western blot. **e** IL-4 conditioned BMDMs were treated with Cis/Dox or Cis-MPs/Dox-MPs for 4 hours, *Cyp* subunits expression was analyzed by real-time PCR. **f** *Cyp1a1*- or *Cyp2j6*-overexpressing BMDMs were stimulated with IL-4 for 12 hours and *Cyp1a1* or *Cyp2j6* expression was determined by real-time PCR. Unless otherwise specified, n=3 biologically independent experiments were performed. Data are presented as mean ± SEM. *P* values were calculated using one-way ANOVA (a-d) and two-tailed unpaired Student’s *t*-tests (f). ***P*<0.01, *****P*<0.0001.


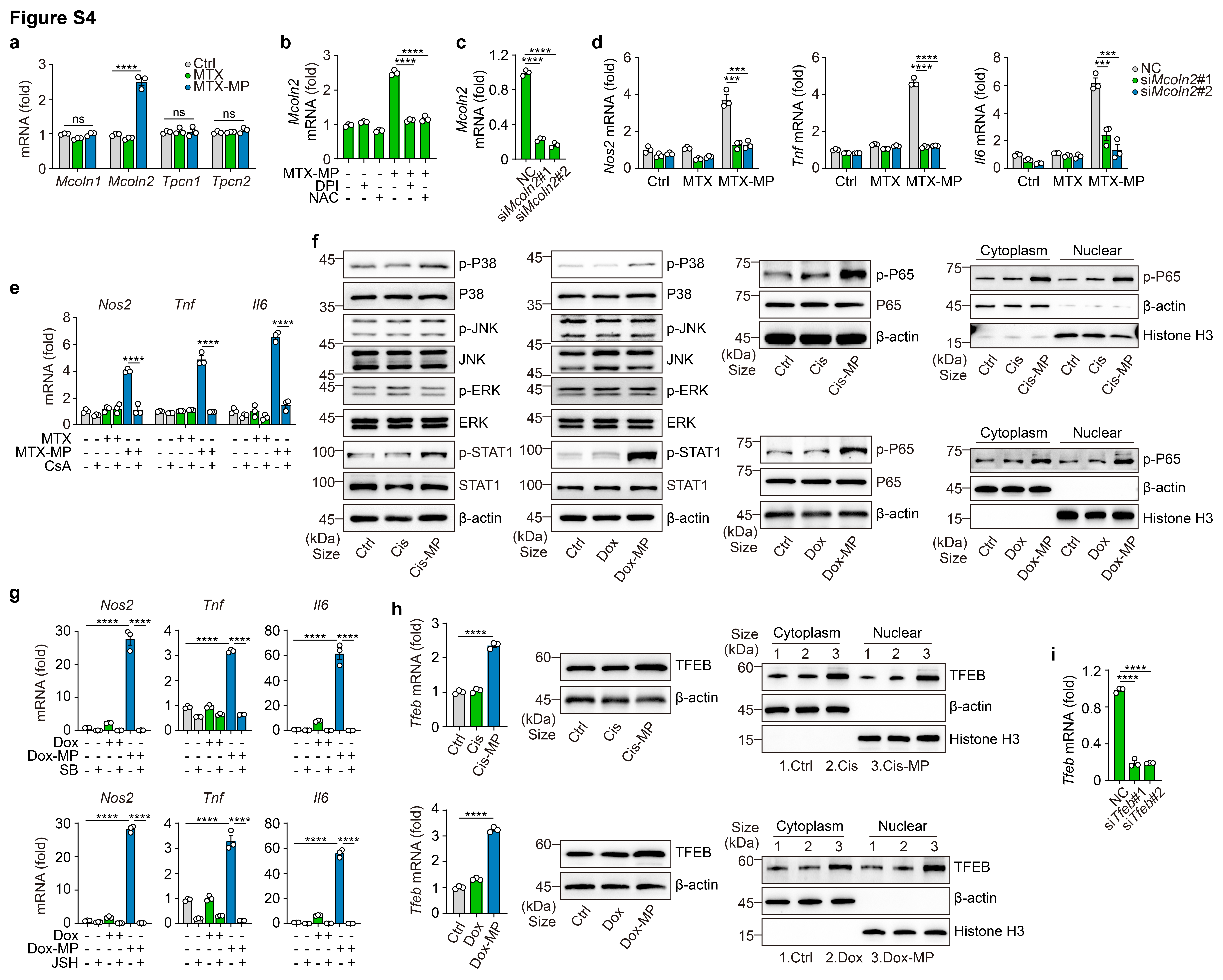


**Fig. S4.** **Lysosomal ROS and pH mediate Ca^2+^ release for macrophage M1 phenotype. a** IL-4 conditioned BMDMs were treated with MTX or MTX-MPs for 12 hours and *Mcoln1*, *Mcoln2*, *Tpcn1* and *Tpcn2* expression was analyzed by real-time PCR. **b** the same as (a), expect that IL-4 conditioned BMDMs were pretreated with 20 mM NAC or 5 μM DPI, *Mcoln2* expression was analyzed by real-time PCR. **c-d** BMDMs were transfected with *Mcoln2* siRNAs for 12 hours and stimulated with IL-4 for 12 hours, *Mcoln2* (c) and *Nos2*, *Tnf*, *Il6* expression (d) was determined by real-time PCR. **e** the same as (d), except that the IL-4 conditioned BMDMs were pretreated with 10 nM CsA. **f** IL-4 conditioned BMDMs were treated with Cis/Dox or Cis-MPs/Dox-MPs for 12 hours, followed by western blot analysis of P38, JNK, ERK, P65 and STAT1. **g** IL-4 conditioned BMDMs were treated with Dox or Dox-MPs alone or combined with SB203580, JSH-23, *Nos2*, *Tnf* and *Il6* expression was determined by real-time PCR. **h** IL-4 conditioned BMDMs were treated with Cis/Dox or Cis-MPs/Dox-MPs for 12 hours and the expression and location of TFEB were analyzed by real-time PCR and western blot. **i** BMDMs were transfected with *Tfeb* siRNAs for 12 hours and stimulated with IL-4 for 12 hours, *Tfeb* expression was determined by real-time PCR. Unless otherwise specified, n=3 biologically independent experiments were performed. Data are presented as mean ± SEM. *P* values were calculated using one-way ANOVA. ****P*<0.001, *****P*<0.0001.


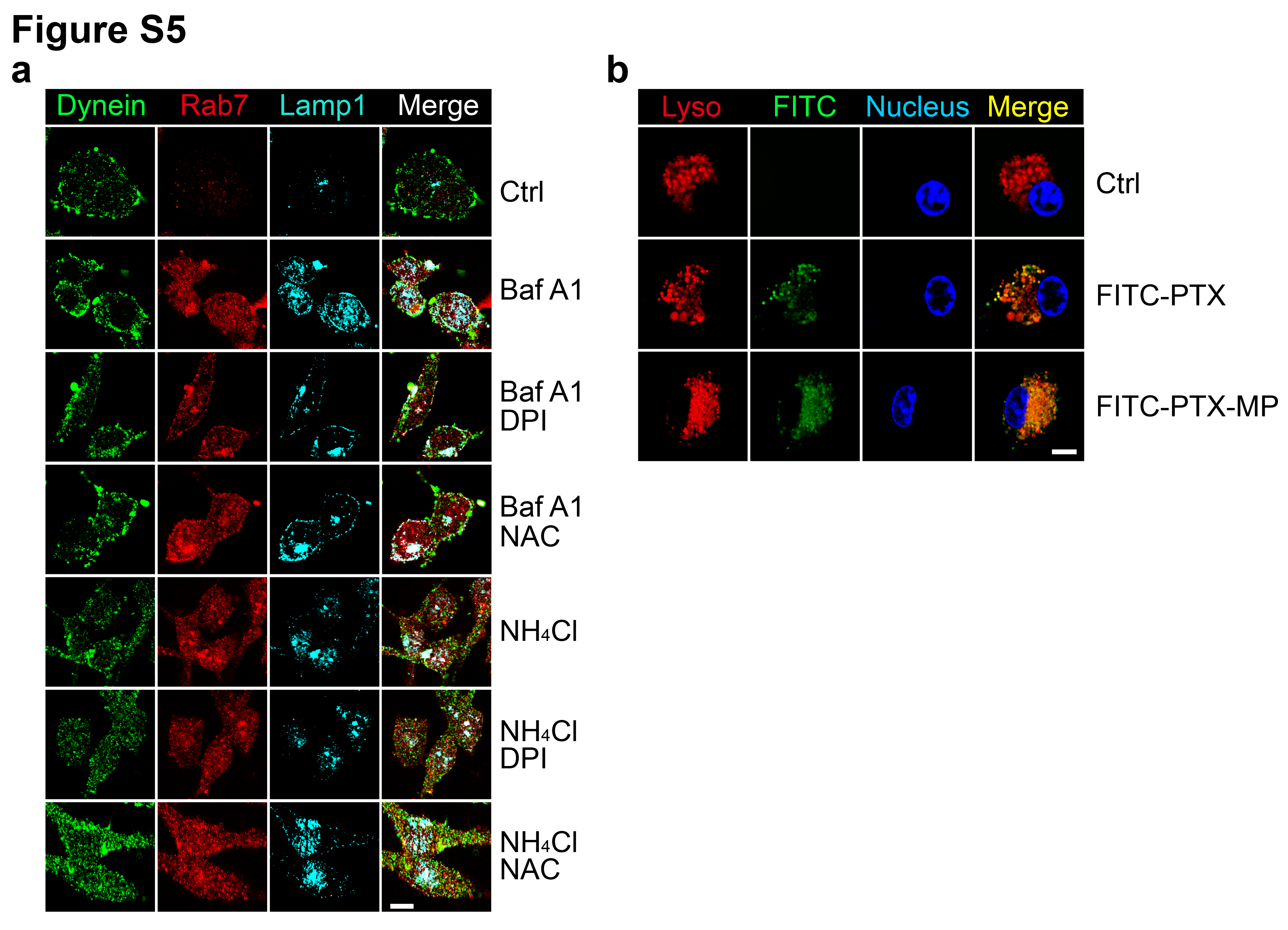


**Fig. S5. Increased lysosomal pH facilitates transfer of lysosomal drugs to the nucleus. a** IL-4 conditioned and NAC or DPI-pretreated BMDMs were treated with MTX-MPs for 24 hours, then treated with bafilomycin A1 or NH_4_Cl for 4 hours and stained with anti-dynein (green), anti-Rab7 (red) and anti-Lamp1 (cyan) antibodies. The BMDMs were observed under a two-photon confocal microscopy. Scale bar, 10 μm. **b** IL-4 conditioned BMDMs were treated with FITC-PTX-MPs for 24 hours and then stained with Lyso-Tracker (red). The BMDMs were observed under a two-photon confocal microscopy. Scale bar, 10 μm.


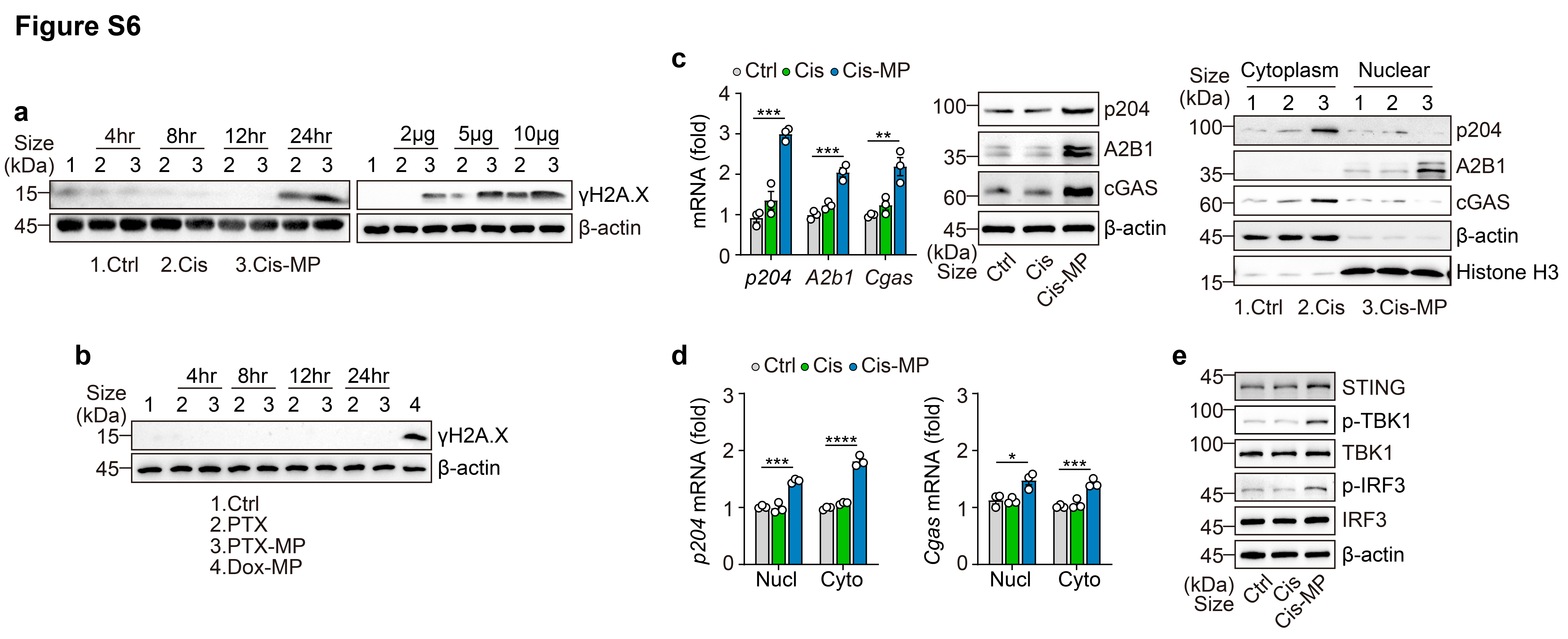


**Fig. S6.** **DNA damage by drug molecules induces type I IFN via hnRNPA2B1-cGAS.** **a** IL-4 conditioned BMDMs were treated with different dose Cis or Cis-MPs for different time and γH2A.X expression was determined by western blot. **b** IL-4 conditioned BMDMs were treated with PTX or PTX-MPs for different time and γH2A.X expression was determined by western blot. **c** IL-4 conditioned BMDMs were treated with Cis or Cis-MPs for 24 hours and p204, hnRNPA2B1 and cGAS expression was determined by real-time PCR (left) and western blot (middle), the location of p204, hnRNPA2B1 and cGAS was analyzed by western blot (right). **d** IL-4 conditioned BMDMs were treated with Cis or Cis-MPs for 24 hours and the nuclear or cytoplasmic *p204* and *Cgas* mRNAs were detected by real-time PCR. **e** IL-4 conditioned BMDMs were treated with Cis or Cis-MPs for 24 hours, followed by western blot analysis of STING, TBK1 and IRF3. Unless otherwise specified, n=3 biologically independent experiments were performed. Data are presented as mean ± SEM. *P* values were calculated using one-way ANOVA. **P*<0.05, ***P*<0.01, ****P*<0.001, *****P*<0.0001.


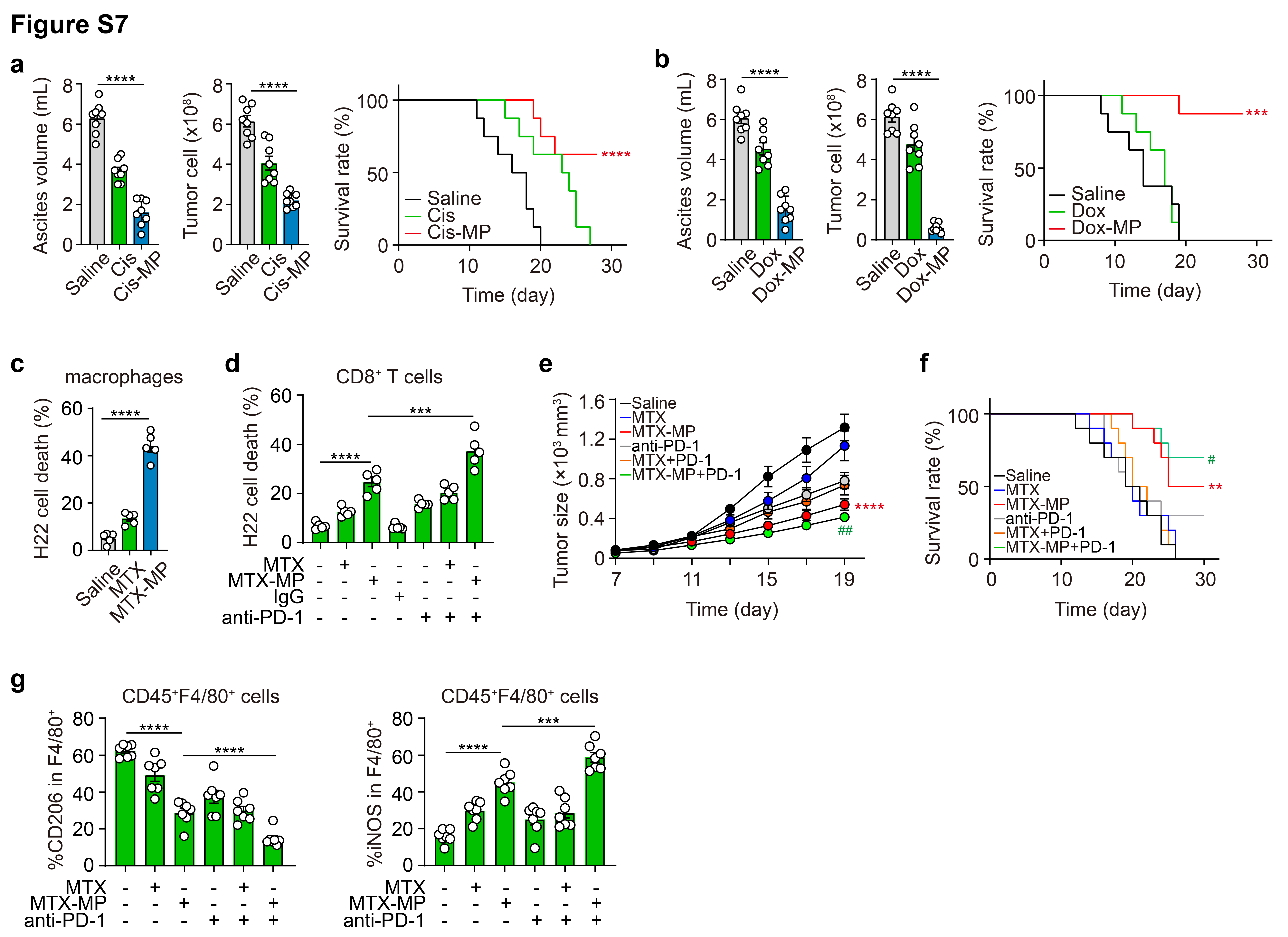


**Fig. S7. Drug-MPs synergize PD-1 blockade to treat malignant ascites. a-b** 1×10^5^ H22 tumor cells were intraperitoneally (i.p.) injected into BALB/c mice. The next day, saline, Cis/Dox (2 µg) or 2×10^6^ Cis-MPs/Dox-MPs (containing ~2 µg Cis/Dox) were i.p. injected into the mice once every day for 15 days. A part of mice was sacrificed and the ascites volume and number of CD45^-^ tumor cells were determined. The long-term survival of tumor-bearing mice was assessed (*n*=8). **c-d** 1×10^5^ H22 tumor cells were i.p. injected into BALB/c mice. The next day, saline, MTX (2 µg) or 2×10^6^ MTX-MPs (containing ~2 µg MTX) were i.p. injected into the mice once every day for 15 days. On day 15, mice were sacrificed. The peritoneal macrophages (E:T=20:1) (c) and CD8^+^ T cells (E:T=10:1) (d) were isolated and incubated with 1×10^5^ H22 tumor cells *in vitro*, the CD45^-^ tumor cell death was detected by flow cytometry. **e-f** Mice were inoculated subcutaneously with B16 cells. The next day, saline, MTX (2 µg) or 2×10^6^ MTX-MPs (containing ~2 µg MTX) were intratumoral injected into the mice once every day for 20 days. At the same time, some mice were i.p. injected with anti-PD-1 neutralizing antibodies (250 µg/mouse) once per 2 days for 20 days. Tumor size and the long-time survival of mice was assessed. (n = 7/10 mice per group). *, MTX-MP vs Saline group #, MTX-MP+anti-PD-1 vs anti-PD-1 group. **g** the same treatment as (e-f), %CD206^+^ in F4/80^+^ cells and %iNOS^+^ in F4/80^+^ cells were analyzed by flow cytometry. Unless otherwise specified, n=3 biologically independent experiments were performed. Data are presented as mean ± SEM. Data are presented as mean ± SEM. *P* values were calculated using one-way ANOVA and a two-sided log-rank (Mantel-Cox) test. ***P*<0.01, ****P*<0.001, *****P*<0.0001, ^#^*P*<0.05, ^##^*P*<0.01.

**Supplementary Table 1. Primer sequence.**

| **GENE** | **PRIMER** | **PRIMER SEQUENCE** |
| --- | --- | --- |
| Mouse *Nos2* | FW | GAGACAGGGAAGTCTGAAGCAC |
|  | RV | CCAGCAGTAGTTGCTCCTCTTC |
| Mouse *Tnf* | FW | GGTGCCTATGTCTCAGCCTCTT |
|  | RV | GCCATAGAACTGATGAGAGGGAG |
| Mouse *Il6* | FW | TAGTCCTTCCTACCCCAATTTCC |
|  | RV | TTGGTCCTTAGCCACTCCTTC |
| Mouse *Il10* | FW | CGGGAAGACAATAACTGCACCC |
|  | RV | CGGTTAGCAGTATGTTGTCCAGC |
| Mouse *Arg1* | FW | CATTGGCTTGCGAGACGTAGAC |
|  | RV | GCTGAAGGTCTCTTCCATCACC |
| Mouse *Il12a* | FW | ACGAGAGTTGCCTGGCTACTAG |
|  | RV | CCTCATAGATGCTACCAAGGCAC |
| Mouse *Il12b* | FW | TTGAACTGGCGTTGGAAGCACG |
|  | RV | CCACCTGTGAGTTCTTCAAAGGC |
| Mouse *Ifnb1* | FW | GCCTTTGCCATCCAAGAGATGC |
|  | RV | ACACTGTCTGCTGGTGGAGTTC |
| Mouse *Mrc1* | FW | GTTCACCTGGAGTGATGGTTCTC |
|  | RV | AGGACATGCCAGGGTCACCTTT |
| Mouse *Cybb* | FW | TGGCGATCTCAGCAAAAGGTGG |
|  | RV | GTACTGTCCCACCTCCATCTTG |
| Mouse *Rac1* | FW | GGACACCATTGAGAAGCTGAAGG |
|  | RV | GTCTTGAGTCCTCGCTGTGTGA |
| Mouse *Rac2* | FW | CTCAGCCAATGTGATGGTGGAC |
|  | RV | CGGACATTCTCATAGGAGGCTG |
| Mouse *Cyp1a1* | FW | CATCACAGACAGCCTCATTGAGC |
|  | RV | CTCCACGAGATAGCAGTTGTGAC |
| Mouse *Cyp1a2* | FW | CATCACAAGTGCCCTGTTCAAGC |
|  | RV | AATGCTCCAGGTGATGGCTGTG |
| Mouse *Cyp1b1* | FW | GCCACTATTACGGACATCTTCGG |
|  | RV | ACAACCTGGTCCAACTCAGCCT |
| Mouse *Cyp2c29* | FW | GCTCTCCTACTCCTGCTGAAGT |
|  | RV | ATGTGGCTCCTGTCTTGCATGC |
| Mouse *Cyp2d9* | FW | GGTCAGAAGTCCTTCATCGCCA |
|  | RV | TGAAGCTGCTCTCAGGATTCCC |
| Mouse *Cyp2d10* | FW | CAGACACCTCTGTGATGCCTTC |
|  | RV | GCATCCTGATGAGGTAAGGGTC |
| Mouse *Cyp2d11* | FW | GGACATTGCTCCACTGCCTTTG |
|  | RV | GACAGTCTCATCTTTCAGCACGG |
| Mouse *Cyp2d12* | FW | CTGTGATGCCTTCACTACTCAGG |
|  | RV | CAGCATCCTGATGAGGTAAGGG |
| Mouse *Cyp2e1* | FW | AGGCTGTCAAGGAGGTGCTACT |
|  | RV | AAAACCTCCGCACGTCCTTCCA |
| Mouse *Cyp2j5* | FW | TGGAACCCAGATGAGCCAAGAG |
|  | RV | AAGAGGTCCAGAGTGGTGCTGA |
| Mouse *Cyp2j6* | FW | GGACCTCTTCTTTGCTGGAACAG |
|  | RV | GCAAGTCTTGCTGCCCTCTTCT |
| Mouse *Cyp2j9* | FW | TCCAGATGAGCCGAGAGACTTC |
|  | RV | AGAAGAGGTCCAGAGTGCTGCA |
| Mouse *Cyp3a11* | FW | ACAGCACTGGTCAGAGCCTGAA |
|  | RV | GAGAGCAAACCTCATGCCAAGG |
| Mouse *Cyp3a13* | FW | TGCTGGCTATGAGACCACAAGC |
|  | RV | GTAGCAGGGTATCATAGGTGGC |
| Mouse *Cyp3a16* | FW | GCAGAGAAAGGCAAGCCTGTTG |
|  | RV | CTTGGCATTCTCCACAAAAGGATC |
| Mouse *Tfeb* | FW | CGCCTGGAGATGACTAACAAGC |
|  | RV | GGCAACTCTTGCTTCACCACCT |
| Mouse *p204* | FW | CCAGTCACCAATACTCCACAGC |
|  | RV | CTCTGAGTGGAGAACAGCACCT |
| Mouse *Hnrnpa2b1* | FW | CGGTGGCAATTTTGGACCAGGA |
|  | RV | CCATAACCAGGGCTACCTCCAA |
| Mouse *Cgas* | FW | TTCCACGAGGAAATCCGCTGAG |
|  | RV | CAGCAGGGCTTCCTGGTTTTTC |
| Mouse *Atp6v0a1* | FW | CTGTTATCCTCGGCATCATCCAC |
|  | RV | CAGGTAGCCAAACAACGAGGAC |
| Mouse *Atp6v0a2* | FW | GCCTCATCTACAACGACTGCTTC |
|  | RV | TGCCTGATGGTGCTGTCATTCC |
| Mouse *Atp6v0a3* | FW | CACAGATGACTAGGGCCCAC |
|  | RV | CAGGGCGGACCGTCTTTATT |
| Mouse *Atp6v0c* | FW | GACTGATGGCATCACCCTCTAC |
|  | RV | CAGGATCATGCCCACGAACAGT |
| Mouse *Atp6v0e* | FW | CTCCTCGTGCCCTGGTTTATCC |
|  | RV | GTGGTCCAAACAGAGGATTGAGC |
| Mouse *Atp6v1a* | FW | GCTGGCTTCTTTCTATGAGCGAG |
|  | RV | GCGTTGCAGAAGTGACTGGATC |
| Mouse *Atp6v1b2* | FW | ATGCGGGGAATCGTGAACG |
|  | RV | AGGCTGGGATAGGTAGTTCCG |
| Mouse *Atp6v1c1* | FW | GGTTGGCTTGTCGGATGAACTG |
|  | RV | TGTCCTCCAGCACATCAGCCAT |
| Mouse *Atp6v1e1* | FW | AAAGGTCGCCTTGTGCAAACGC |
|  | RV | CTCTGAGGACTTTGAGCCTTGC |
| Mouse *Atp6v1f* | FW | GGGAGCTAAACAAGAACCGCCA |
|  | RV | CCATCTCTGCGATGTACTGGTTG |
| Mouse *Atp6v1g1* | FW | CAGAGGGAGAAGGAGTTCAAGG |
|  | RV | GTTCTGGAGGACGGTCATCTTC |
| Mouse *Atp6v1h* | FW | GTTGCTGCTCACGATGTTGGAG |
|  | RV | TGTAGCGAACCTGCTGGTCTTC |
| Mouse *Mcoln1* | FW | ACCATCTCGGGGACTGTCAT |
|  | RV | CAGGTAGCGAATGACACCGA |
| Mouse *Mcoln2* | FW | GCATTCTGGTGTGGCTGTTC |
|  | RV | GGTGTGGTAAGAGTCGGTG |
| Mouse *Tpcn1* | FW | CCCTGGAGTTACCTCGTGTTTC |
|  | RV | GAATGCCGTGACCGAGAAATCG |
| Mouse *Tpcn2* | FW | CATCCACCTGTGTCTCTTCACC |
|  | RV | GTGAGGTCAGTGCTTCTGGAAG |
| Mouse *Actb* | FW | GGCTGTATTCCCCTCCATCG |
|  | RV | CCAGTTGGTAACAATGCCATGT |

Supplementary Table 2. siRNA Sequence.

| **GENE** | **SIRNA** | **SEQUENCE** |
| --- | --- | --- |
| mouse *Cybb* | siRNA#1 | GCTGAATGTCTTCCTCTTT |
|  | siRNA#2 | CCATGGAGCTGAACGAATT |
| mouse *Mcoln2* | siRNA#1 | GCAGTTCATTCCCGAGAGA |
|  | siRNA#2 | GCTTGAAGGTCTGTAAGCA |
| mouse *Stat1* | siRNA#1 | CTGTGATGTTAGATAAACA |
|  | siRNA#2 | GCAGCACAACATACGGAAA |
| mouse *Tfeb* | siRNA#1 | GCAGGCTGTCATGCATTAT |
|  | siRNA#2 | CCATGGCCATGCTACATAT |
| mouse *Rab7* | siRNA#1 | CCATCAAACTGGACAAGAA |
|  | siRNA#2 | GGAAGAAAGTGTTGCTGAA |
| mouse *Dync1h1* | siRNA#1 | GAAATCAACTTGCCCGATA |
|  | siRNA#2 | CCACGTGCCTGTTGTATAT |
| mouse *Hnrnpa2b1* | siRNA#1 | TGGAAGGAGCAGATATTAA |
|  | siRNA#2 | GAAGTGGAAGTTACAATGA |
| mouse *Cgas* | siRNA#1 | GGCCGAGACGGTGAATAAA |
|  | siRNA#2 | GGATTGAGCTACAAGAATA |

**Supplementary Table 3. Western Blot Antibodies**

| **ANTIBODIES** | **SOURCE** | **IDENTIFIER** |
| --- | --- | --- |
| anti-iNOS | Cell Signaling Technology | D6B6S#13120 |
| anti-arginase1 | Cell Signaling Technology | D4E3M#93668 |
| anti-phospho-P38-Thr180/Tyr-182 | Cell Signaling Technology | 3D7#9215 |
| anti-P38 | Cell Signaling Technology | D13E1#8690 |
| anti-phospho-ERK-Thr202/Tyr204 | Cell Signaling Technology | 194G2#4377S |
| anti-ERK | Cell Signaling Technology | 137F5#4695S |
| anti-phospho-JNK-Thr183/Tyr185 | Cell Signaling Technology | 9251S |
| anti-JNK | Cell Signaling Technology | 9252S |
| anti-phospho-STAT1-Tyr701 | Cell Signaling Technology | 58D6#9167 |
| anti-STAT1 | Cell Signaling Technology | 9H2#9176 |
| anti-phospho-TBK1-Ser172 | Cell Signaling Technology | D52C2#5483 |
| anti-TBK1 | Cell Signaling Technology | D1B4#3504 |
| anti-NF-κB p65 | Cell Signaling Technology | D14E12#8242 |
| anti-phospho-NF-κB p65-Ser536 | Cell Signaling Technology | 93H1#3033 |
| anti-phospho-Histone-H2A.X-Ser139 | Cell Signaling Technology | 2577 |
| anti-phospho-IRF3-Ser396 | Cell Signaling Technology | 4D4G#4947 |
| anti-cGAS | Cell Signaling Technology | D1D3G#66546 |
| anti-β-actin | Cell Signaling Technology | 8H10D10#3700 |
| anti-Histone H3 | Cell Signaling Technology | D1H2#4499 |
| anti-NOX2/gp91^phox^ | Abcam | ab129068 |
| anti-Rac2 | Abcam | ab154711 |
| anti-TFEB | Abcam | ab264421 |
| anti-p204 | Abcam | ab228512 |
| anti-hnRNPA2B1 | Abcam | ab183654 |
| anti-STING | Abcam | ab198951 |
| anti-IRF3 | Abcam | ab68481 |

**Supplementary Table 4. Fluorescent Antibodies**

| **ANTIBODIES** | **SOURCE** | **IDENTIFIER** |
| --- | --- | --- |
| PerCP anti-human CD45 | Biolegend | 304026, HI30 |
| PE anti-human CD163 | Biolegend | 333606, GHI/61 |
| FITC anti-mouse CD80 | Biolegend | 104706, 16-10A1 |
| PE anti-mouse CD86 | Biolegend | 105008, GL-1 |
| FITC anti-mouse PD-1 (CD279) | Biolegend | 135214, 29F.1A12 |
| FITC anti-mouse F4/80 | Biolegend | 123108, BM8 |
| APC anti-mouse F4/80 | Biolegend | 123116, BM8 |
| PerCP/Cyanine anti-mouse CD8α | Biolegend | 100734, 53-6.7 |
| PE anti-mouse CD107a | Biolegend | 121612, 1D4B |
| PerCP/Cyanine5.5 anti-mouse CD45 | Biolegend | 103132, 30-F11 |
| APC anti-human CD68 | Biolegend | 333810, Y1/82A |
| FITC anti-human CD206 | Biolegend | 321104, 15-2 |
| PE anti-human iNOS | Biolegend | 696806, W16030C |
| FITC anti-mouse CD206 | Biolegend | 141704, C068C2 |
