# Supplementary material for: Chemo-drugs in cell microparticles reset antitumor activity of macrophages by activating lysosomal P450 and nuclear hnRNPA2B1
Source: Signal Transduct Target Ther. 2023 Jan 20;8:22. doi: 10.1038/s41392-022-01212-7 (PMC9852455; doi:10.1038/s41392-022-01212-7)

## Full unedited and uncropped gels

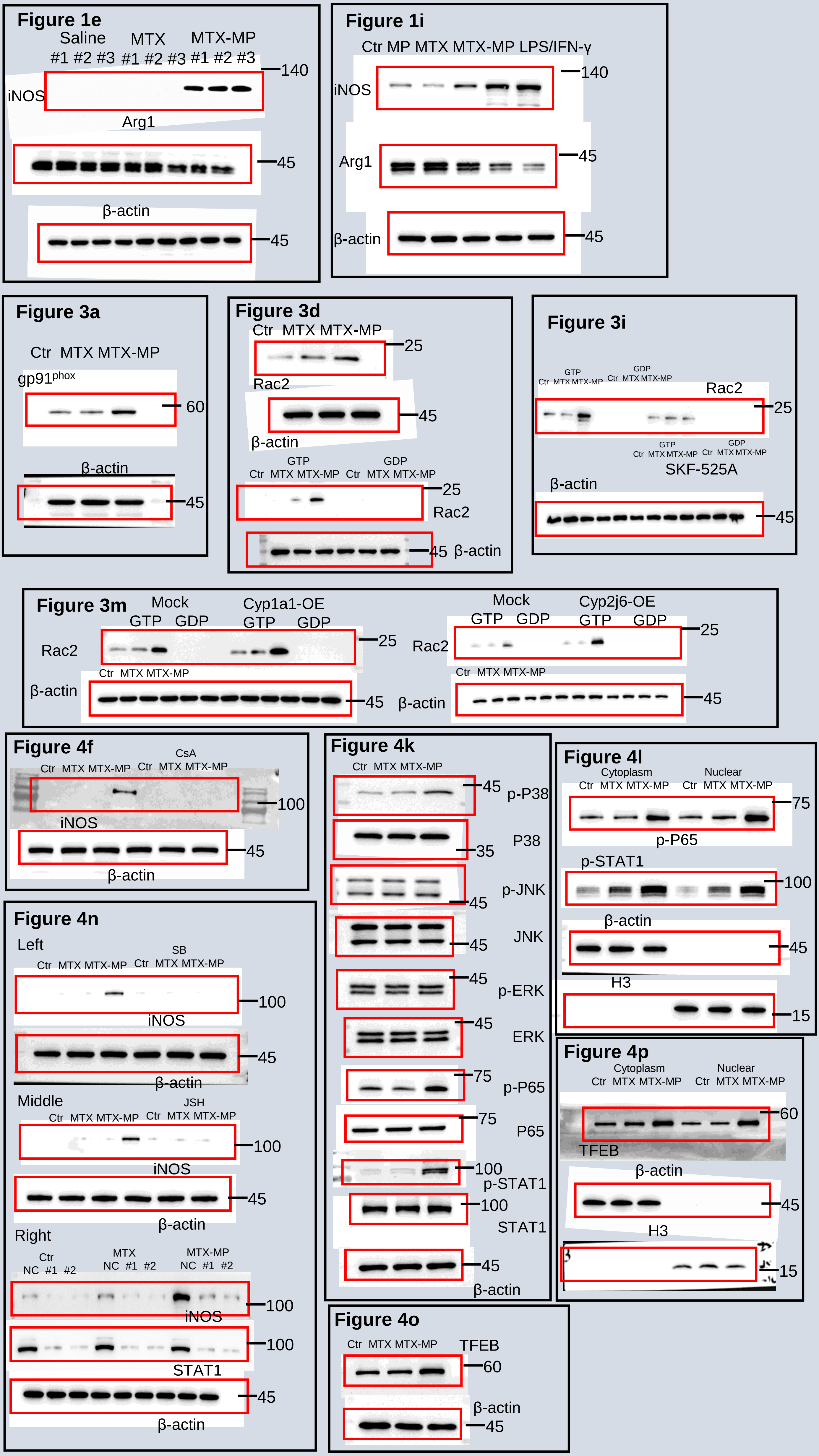

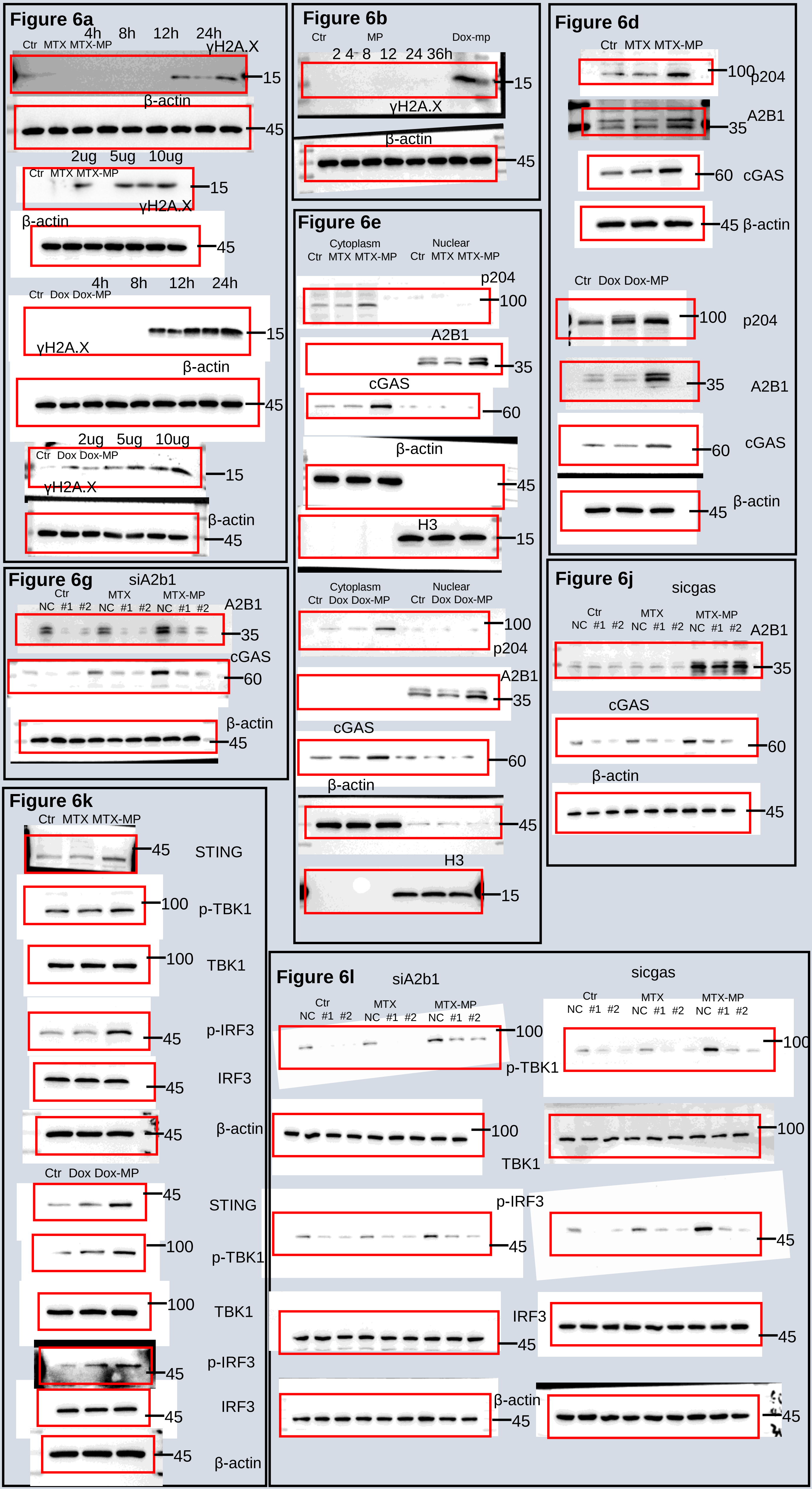
Figure 6a
4h 8h 12h 24h
Ctr MTX MTX-MP
γH2A.X
15
β-actin
45
2ug 5ug 10ug
Ctr MTX MTX-MP
15
γH2A.X
β-actin
45
4h 8h 12h 24h
Ctr Dox Dox-MP
15
γH2A.X
β-actin
45
2ug 5ug 10ug
Ctr Dox Dox-MP
15
γH2A.X
β-actin
45
Figure 6b
Ctr MP Dox-mp
2 4 8 12 24 36h
15
γH2A.X
β-actin
45
Figure 6d
Ctr MTX MTX-MP
100 p204
A2B1
35
60 cGAS
45 β-actin
Ctr Dox Dox-MP
100 p204
35 A2B1
60 cGAS
β-actin
45
Figure 6e
Cytoplasm Nuclear
Ctr MTX MTX-MP Ctr MTX MTX-MP
p204
100
A2B1
35
cGAS
60
β-actin
45
H3
15
Cytoplasm Nuclear
Ctr Dox Dox-MP Ctr Dox Dox-MP
100
p204
A2B1
35
cGAS
60
β-actin
45
H3
15
Figure 6g
siA2b1
Ctr MTX MTX-MP
NC #1 #2 NC #1 #2 NC #1 #2
A2B1
35
cGAS
60
β-actin
45
Figure 6j
sicgas
Ctr MTX MTX-MP
NC #1 #2 NC #1 #2 NC #1 #2
A2B1
35
cGAS
60
β-actin
45
Figure 6k
Ctr MTX MTX-MP
45 STING
100 p-TBK1
100 TBK1
45 p-IRF3
45 IRF3
45 β-actin
Ctr Dox Dox-MP
45 STING
100 p-TBK1
100 TBK1
45 p-IRF3
45 IRF3
45 β-actin
Figure 6l
siA2b1
Ctr MTX MTX-MP
NC #1 #2 NC #1 #2 NC #1 #2
sicgas
Ctr MTX MTX-MP
NC #1 #2 NC #1 #2 NC #1 #2
100
p-TBK1
100
100
TBK1
100
p-IRF3
45
45
IRF3
45
45
β-actin
45
45

## Full unedited and uncropped gels

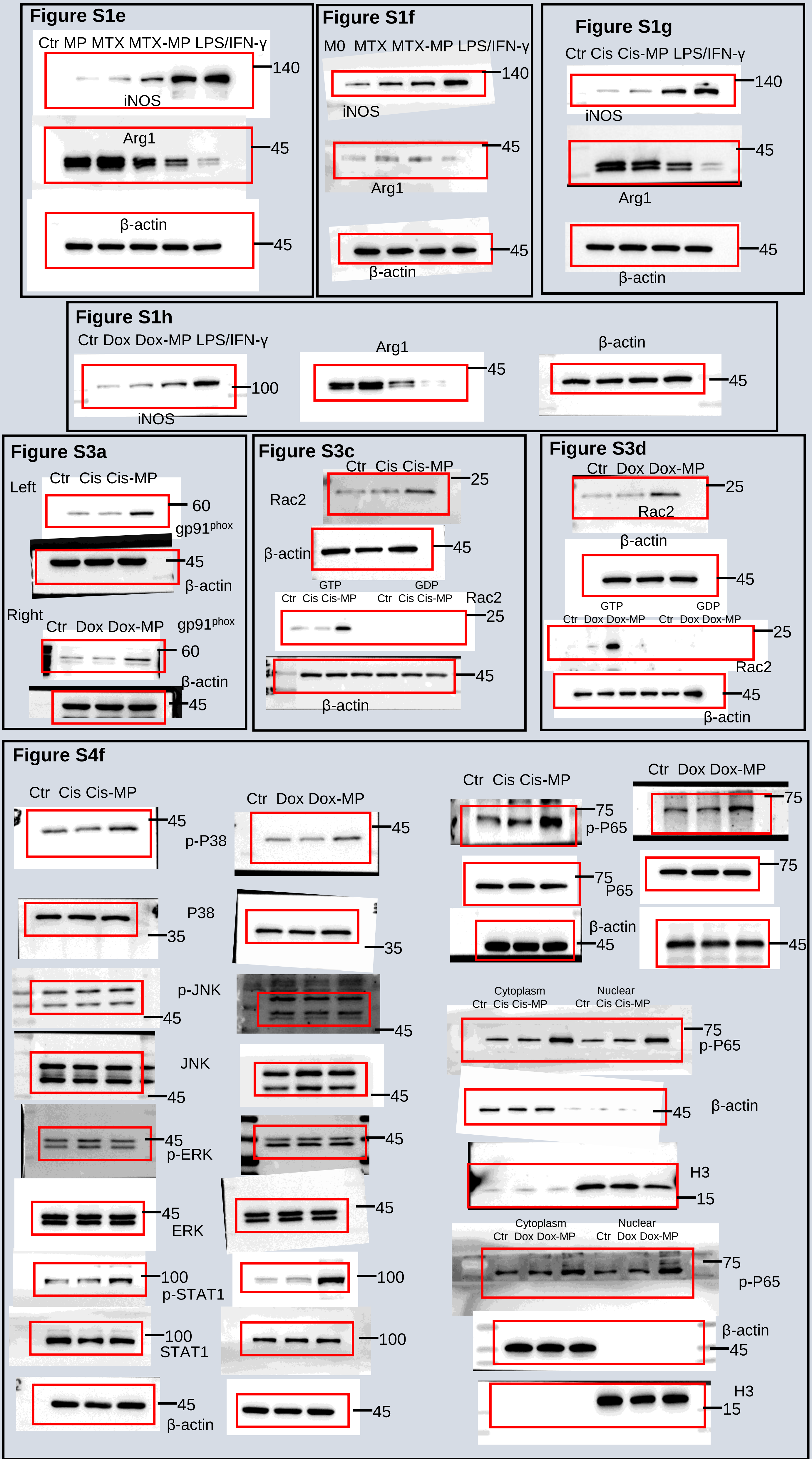

**Full unedited and uncropped gels**

## Figure S4h

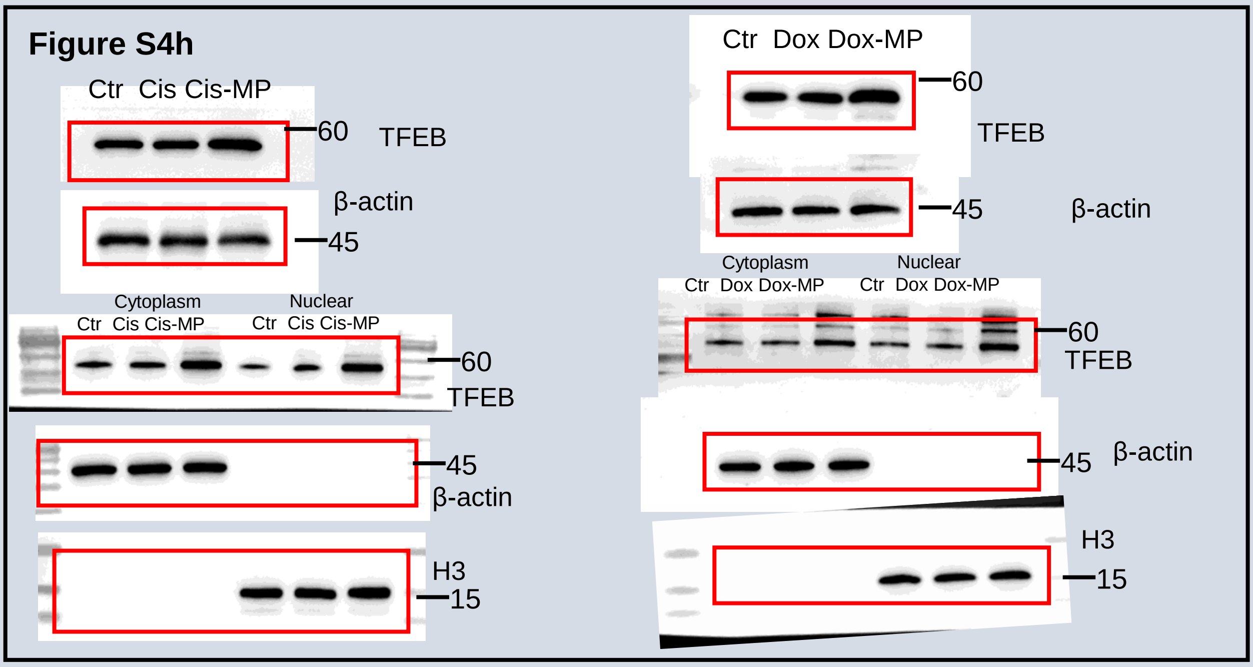


## Figure S6a

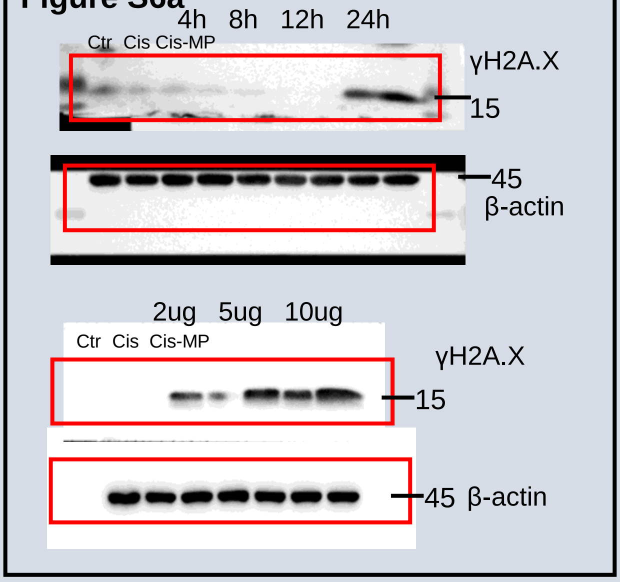


## Figure S6b

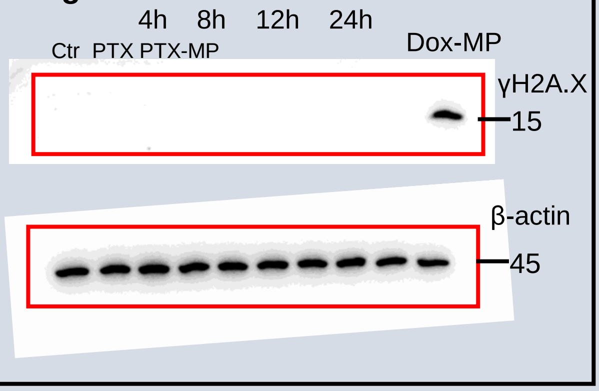


## Figure S6c

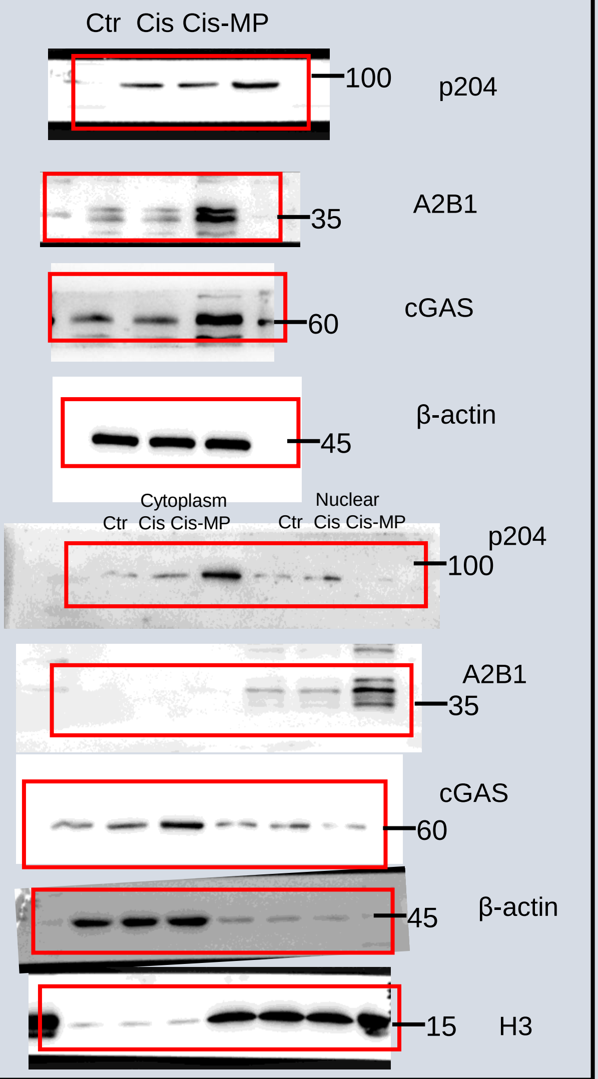


## Figure S6e

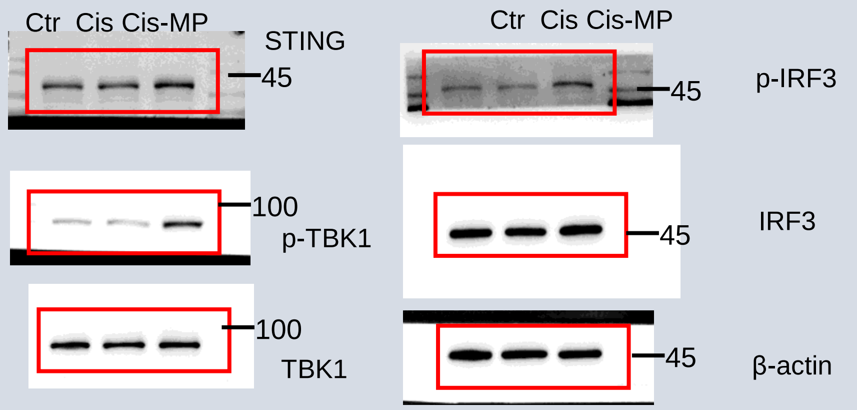

Supplement: Supplementary file 2 — Supplemental information [file 41392_2022_1212_MOESM2_ESM.pdf]
